# Ensemble-based modeling and rigidity decomposition of allosteric interaction networks and communication pathways in cyclin-dependent kinases: Differentiating kinase clients of the Hsp90-Cdc37 chaperone

**DOI:** 10.1371/journal.pone.0186089

**Published:** 2017-11-02

**Authors:** Gabrielle Stetz, Amanda Tse, Gennady M. Verkhivker

**Affiliations:** 1 Department of Computational and Data Sciences, Schmid College of Science and Technology, Chapman University, Orange, California, United States of America; 2 Department of Biomedical and Pharmaceutical Sciences, Chapman University School of Pharmacy, Irvine, California, United States of America; George Mason University, UNITED STATES

## Abstract

The overarching goal of delineating molecular principles underlying differentiation of protein kinase clients and chaperone-based modulation of kinase activity is fundamental to understanding activity of many oncogenic kinases that require chaperoning of Hsp70 and Hsp90 systems to attain a functionally competent active form. Despite structural similarities and common activation mechanisms shared by cyclin-dependent kinase (CDK) proteins, members of this family can exhibit vastly different chaperone preferences. The molecular determinants underlying chaperone dependencies of protein kinases are not fully understood as structurally similar kinases may often elicit distinct regulatory responses to the chaperone. The regulatory divergences observed for members of CDK family are of particular interest as functional diversification among these kinases may be related to variations in chaperone dependencies and can be exploited in drug discovery of personalized therapeutic agents. In this work, we report the results of a computational investigation of several members of CDK family (CDK5, CDK6, CDK9) that represented a broad repertoire of chaperone dependencies—from nonclient CDK5, to weak client CDK6, and strong client CDK9. By using molecular simulations of multiple crystal structures we characterized conformational ensembles and collective dynamics of CDK proteins. We found that the elevated dynamics of CDK9 can trigger imbalances in cooperative collective motions and reduce stability of the active fold, thus creating a cascade of favorable conditions for chaperone intervention. The ensemble-based modeling of residue interaction networks and community analysis determined how differences in modularity of allosteric networks and topography of communication pathways can be linked with the client status of CDK proteins. This analysis unveiled depleted modularity of the allosteric network in CDK9 that alters distribution of communication pathways and leads to impaired signaling in the client kinase. According to our results, these network features may uniquely define chaperone dependencies of CDK clients. The perturbation response scanning and rigidity decomposition approaches identified regulatory hotspots that mediate differences in stability and cooperativity of allosteric interaction networks in the CDK structures. By combining these synergistic approaches, our study revealed dynamic and network signatures that can differentiate kinase clients and rationalize subtle divergences in the activation mechanisms of CDK family members. The therapeutic implications of these results are illustrated by identifying structural hotspots of pathogenic mutations that preferentially target regions of the increased flexibility to enable modulation of activation changes. Our study offers a network-based perspective on dynamic kinase mechanisms and drug design by unravelling relationships between protein kinase dynamics, allosteric communications and chaperone dependencies.

## Introduction

Protein kinases govern functional processes in cellular networks by acting as dynamic molecular switches that fluctuate between ensembles of the inactive and active forms [1–7]. Structural mechanisms regulating dynamic kinase equilibrium operate under allosteric control, in which phosphorylation of the activation loops and/or binding partners trigger global rearrangements that stabilize a catalytically competent kinase form [8–12]. Conformational changes in the kinase catalytic domain are orchestrated by allosteric coupling of the regulatory regions: the αC-helix, the catalytic HRD motif, the DFG-Asp motif (DFG-Asp in, active; DFG-Asp out, inactive), and the activation loop (A-loop open, active; A-loop closed, inactive). The HRD histidine is conserved through all eukaryotic and eukaryotic-like kinases, serving as an integrating scaffold which binds to the regulatory DFG motif. Structural and evolutionary analyses demonstrated that HxD-histidine is a focal site of the kinase core for various catalytic, regulatory and substrate-binding regions, because of its strategic position and multiple conserved interactions with other functional residues [13–15]. Conformational strain in the catalytically important HRD motif was found to be a common feature of the active conformation for many kinases, and may have evolved to enable allosteric control of catalytic activity [13]. The superposition of the HxD motifs in multiple crystal structures of activated eukaryotic protein kinase (EPK) indicated a high degree of structural conservation in activated protein kinases, as this residue is irreplaceable for the maintenance of kinase activity [14,15]. The HRD arginine is conserved only in the eukaryotic kinases, and the HRD motif is often referred to as an HxD motif. Protein kinases with arginine at this position typically require phosphorylation of the A-loop, and the HRD arginine integrates the catalytic loop, phosphorylation site and the magnesium-binding loop.

Structural studies of protein kinases have shown that the inactive kinase conformations may fall into a number of classes in which certain key features of the inactivation mechanism are conserved [16–18]. A common regulatory theme for a large class of protein kinases is based on sharing an autoinhibitory inactive conformation that had been initially discovered in cyclin-dependent kinases (CDK) and the Src kinases, but was later observed on different evolutionary branches of the human kinome [19]. This inactive kinase state, which is termed as Cdk/Src conformation, is characterized by a structural arrangement in which the regulatory αC-helix is displaced outwards the N-terminal lobe adopting a αC-out conformation that inhibits the formation of the active enzyme form. The growing wealth of structural knowledge about conformational states of the kinase catalytic domain, regulatory assemblies and kinase complexes with inhibitors has dramatically advanced out understanding of the molecular determinants underlying kinase structure, function and binding [20–25]. The large pool of kinase structures has shown that the kinase equilibrium operates not as a simple binary switch but rather reflects thermal fluctuations on a complex conformational landscape that is dominated by major basins of the inactive (DFG-out/αC-helix-in), the Cdk/Src-like inactive (DFG-in/αC-helix-out) and the active kinase form (DFG-in/αC-helix-in). Allosteric coupling between the regulatory DFG moiety and the αC-helix controls a dynamic equilibrium between the inactive and active kinase forms. The inter-lobe dynamics and kinase activation are also tightly linked with the structural assembly of two intramolecular hydrophobic networks forming regulatory spine (R-spine) and a catalytic spine (C-spine) [10–12].

CDK proteins is a group of serine/threonine kinases with multiple isoforms that are quintessential of kinase-targeted drug discovery with more than 20 inhibitors in clinical trials, and the first FDA-approved drug palbociclib [26–35]. This kinase subfamily had achieved its initial therapeutic prominence and attention from pharmaceutical industry due to a critical role of CDKs in cell cycle control, but was subsequently implicated in other functions—from epigenetics (CDK2, CDK4), to control of neuronal activity (CDK5), and regulation of gene transcription (CDK7, CDK9) [29,30]. A large number of CDK crystal structures in different forms along with a significant body of biochemical and cell-based studies have provided significant insights into diverse functions of these enzymes that can be regulated through binding of cyclins and phosphorylation of the activation loop (often termed as T-loop) [28–31]. While cyclin A binding can induce conformational changes and activation of CDK2 [36–39], crystal structures of CDK4-cyclin D complexes demonstrated that even a combination of cyclin D binding and T-loop phosphorylation in CDK4 is not sufficient for the kinase domain to attain an active conformation [40,41]. At the same time, Cdk5 is activated by the non-cyclin proteins Cdk5R1 (p35, p25) or Cdk5R2 (p39), and phosphorylation in the T-loop is not required for its activation [33, 42–44]. Several members of the CDK family, including CDK9, are also involved in transcription, where with CDK9/cyclin forming the core of the positive transcription elongation factor b (P-TEFb) [45]. The crystal structures of CDK2-cyclin A [36,37], CDK4-cyclin D [40,41], CDK5/p25 [44], CDK1-cyclin B [46], CDK6/V-cyclin [47], and CDK9/cyclin T complexes [48–50] have revealed noticeable differences, showing that the relative orientation and position of the cyclin in CDK-bound complexes can vary from large binding interface in CDK2 and CDK5 proteins to a much smaller interface and more open structure in the CDK4 and CDK9 complexes. CDK9 is a transcriptional CDK that autophosphorylates at several sites and is characterized by an extended C-terminal tail as compared to canonical CDKs fold and this segment determines the CDK9 kinetic pathway [49]. The smaller interface between CDK9 and cyclin T produces weaker binding as compared to a much stronger association between CDK2 and cyclin A [48]. Structural variations in the binding interfaces can raise the susceptibility of CDK9/cyclinT1 for recruitment of additional binding partners and formation of supramolecular complexes. The crystal structure of the multiprotein Tat-AFF4- P-TEFb complex that contained virus-encoded transcription factor HIV-1 Tat, Cyclin T1, Cdk9 domain, and AFF4 scaffolding protein revealed how CDK9 kinase subunit and cyclin T1 can be engaged in the supramolecular elongation complex [51–53]. These illuminating studies offered compelling evidence that structural and functional plasticity of CDK9 may allow for diverse and complex assemblies regulating activity of multiple interacting proteins.

Among notable divergences in the regulatory mechanisms of CDK proteins are radically different dependencies on the Hsp90 chaperone machinery that is required for some members of CDK family for fold maturation and stabilization of the active conformation. The 90 kDa heat-shock proteins Hsp90s manage late stages of conformational development, maturation and folding for a wide array of protein client substrates, including protein kinases [54–61]. The Hsp90 cochaperones can facilitate client recruitment and modulate progression of the Hsp90-ATPase cycle by stabilizing specific chaperone states [62–65]. Cdc37 is a kinase-specific cochaperone that in coordination with Hsp90 can facilitate conformational maturation and acquisition of functional states for a large and diverse clientele of protein kinases [66, 67]. The biochemical and functional studies indicated that the Hsp90-Cdc37 chaperone could distinguish kinase clients by recognizing their conformational instability in the N-terminal lobe and promoting formation of transient chaperone-client intermediates, while rejecting stable native folds of nonclient kinases [68–71]. A high-throughput study of the Hsp90-client interactions provided a first quantitative analysis of the Hsp90-client interactions and unveiled key principles of substrate recognition [72]. The observed correlation between the Hsp90-kinase and Cdc37-kinase interactions demonstrated that the chaperone machinery operates in a cooperative manner to recognize and support maturation of client kinases. Moreover, the strength of the interactions between Hsp90 and kinases strongly correlated with the thermal instability of the kinase domain, suggesting that kinases with dynamic native folds may be intrinsically predisposed for stronger association with the chaperone system. Based on a quantitative thermodynamic analysis of chaperone dependencies, this pioneering study presented the first classification of the kinases into nonclients, weak and strong client of the Hsp90-Cdc7chaperone [72]. A large scale chemical proteomic profiling identified a total of 288 protein kinase clients, among which 98 were downregulated upon geldanamycin treatment including 44 previously confirmed and 51 down-regulated kinases not previously implicated in Hsp90 regulation [73]. HX-MS studies of structurally similar client and nonclient kinase chimeras suggested that kinase dependence on the Hsp90-Cdc37 chaperone is associated with the degree of unfolding cooperativity and client compactness that favor exposure of the key regulatory regions in the catalytic domain [74]. Functional assays and computational analysis of the wild type c-Src kinase and the oncogenic mutant variant v-Src demonstrated that drastic difference in the Hsp90-dependence of these proteins can be determined by the intrinsic dynamic preferences of the mutant client [75]. Despite significant insights and recent breakthroughs in biochemical characterization of the Hsp90-Cdc37 interactions with a diverse kinase clientele, structural details of client recognition and binding with the chaperone remained unknown until recently. The latest cryo—electron microscopy structure of the Hsp90-Cdc37-Cdk4 kinase complex marked an important milestone in understanding molecular basis of kinase recruitment by the chaperone, showing that the dynamic landscapes of client kinases are highly heterogeneous and can be readily converted into partially unfolded states by the chaperone [76].

Structure-functional characterization of the kinase clientele also indicated that the Hsp90-Cdc37 machinery often favors kinases that assume a Cdk/Src-like inactive state and engage in activation-promoting interactions with binding partners to achieve their functional form (S1 Fig) [72]. This regulatory mechanism is shared by members of the CDK family that commonly feature a Cdk/Src-like conformation in their inactive form and exploit interactions with cyclins to attain catalytically competent conformations. Strikingly, significant differences in the chaperone dependencies were observed for CDK proteins, where several members of this family (CDK1, CDK2, CDK5) were identified as nonclients of the chaperone, CDK6 emerged as a weak client, while other prominent members of this family (CDK4, CDK7, and CDK9) appeared to be strong clients of the Hsp90-Cdc37 chaperone [72,77]. The molecular determinants underlying chaperone dependencies of protein kinases are not fully understood as minor structural differences between these kinases are difficult to relate with the radical switching in regulatory responses to the chaperone. The regulatory divergences observed for CDK proteins are of particular interest as functional diversification among members of this family may be linked with variations in chaperone dependencies. Delineating molecular principles underlying differentiation of protein kinase clients and chaperone modulation of kinase activity is also of significant therapeutic interest, as the Hsp90 system often recruits kinases that are abnormally activated by mutations in cancer cells.

Computational studies have elucidated many aspects of CDK structure, dynamics and binding. Molecular dynamics (MD) simulations and coarse-grained elastic models [78, 79] have investigated collective motions in different CDK2 structures [80–82]. Simulations of conformational transitions between the open and closed states of CDK2 showed that activation may be regulated by the αC-helix and T-loop regions, which undergo large structural changes during remodeling of the kinase domain [83]. MD simulations of the CDK2-Cyclin A/substrate complex analyzed the role of the phosphorylated T160 site on dynamics and substrate binding, confirming its important role for substrate recognition and thermal stability [84]. Normal mode analysis and enhanced sampling simulations of CDK2 and CDK4 complexes showed that the active conformation is the most stable for the CDK2-cyclin A, while a dynamic equilibrium between open and closed states was observed in the CDK4-cyclin D1 complexes [85]. Accelerated MD simulations examined conformational landscapes of CDK2 kinase in the apo form and in the complex with an allosteric inhibitor [86]. MD simulations of the Tat/Cyclin T1/CDK9 complex presented the first detailed study of supramolecular assemblies that involve transcriptional CDK, revealing how presence of multiple binding partners may promote structural environment favoring formation of the active state [87,88]. These studies exemplified a considerable progress in characterizing conformational ensembles and transitions in CDK proteins [89].

In this work, we report the results of a computational investigation of several members of CDK family (CDK5, CDK6, CDK9) that represented a broad repertoire of chaperone dependencies—from nonclient CDK5, to weak client CDK6, and strong client CDK9. The principal hypotheses and conceptual framework of our study are based on the premise that mechanisms of CDK regulation and activation by binding partners are ultimately determined by the dynamic conformational landscapes and organization of the allosteric interaction networks in CDK structures. In this formulation, binding of activating partners and phosphorylation could promote thermodynamic shifts between allosteric states by modulating structure and stability of the interaction networks. These phenomena can be conveniently described using an ensemble-based model of allosteric interactions [90–93] and graph-based network analysis of allosteric interactions and communications in protein structures [94–101]. We investigate how differences in the conformational dynamics, energetics, allosteric interaction networks and communication pathways in CDK proteins may be linked to their unique chaperone dependencies and regulatory mechanisms. Discrete molecular dynamics (DMD) was used to simulate multiple crystal structures of CDK proteins in the unbound and complexed states. Principal component analysis of conformational ensembles and elastic network modeling of multiple crystal structures deciphered differences in functional motions and collective dynamics of CDK structures. The ensemble-based network community analysis and modeling of communication pathways explored organization of the residue interaction networks and determined functional role of allosteric hotspots in kinase regulation. Network analysis was also integrated with the perturbation response scanning and rigidity decomposition approaches to probe how structural stability and allosteric cooperativity are connected with kinase propensities for chaperone binding. By showcasing a panel of CDK proteins that span the full repertoire of chaperone dependencies, we identified dynamic and network signatures that can differentiate kinase clients and rationalize subtle divergences in the activation mechanisms of CDK family members.

## Results and discussion

### Molecular simulations and collective dynamics of the CDK structures expose the elevated mobility of the client kinase

**Fig 1:**
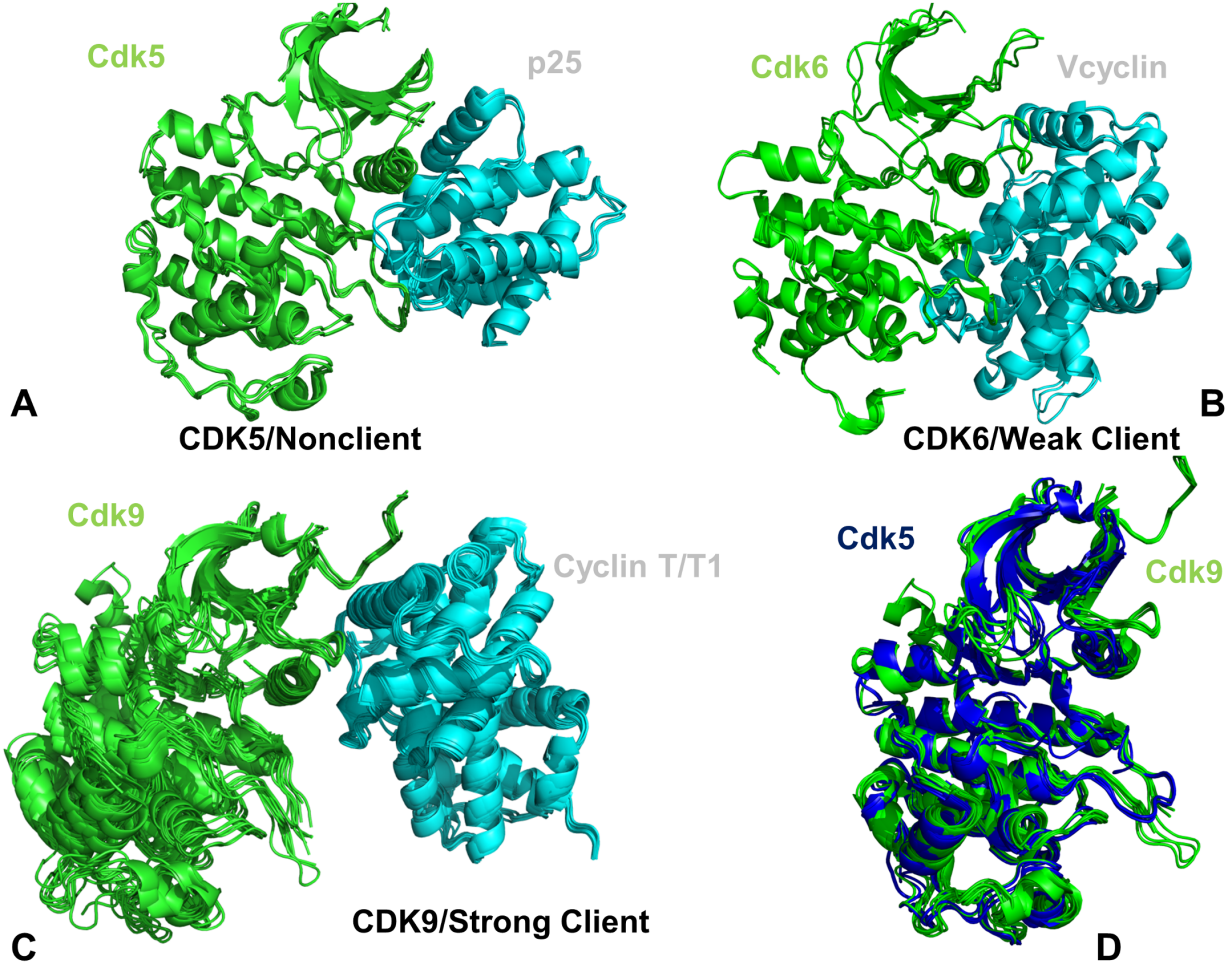
Crystal structures of the CDK5-p25, CDK6/V-cyclin and CDK9-cyclin T complexes. (A) The crystal structures of the panel of CDK5-p25 complexes (pdb id 14H4L, 1UNG, 1UNH, 1UNL, 3O0G, 4AU8). (B) The crystal structures of CDK6/V-cyclin complexes (pdb id 2EUF, 2F2C) and CDK6 complexes withy inhibitors (pdb id 5L2I, 5L2S, and 5L2T). (C) The crystal structures of CDK9-cyclin T/T1 complexes (pdb id 3BLH, 3BLQ, 3BLR, 3LQ5, 3MIA, 3TN8, 3TNH, 4BCF, 4BCH, 4BCI, 4BCJ, 4EC8, 4EC9, 4IMY). The catalytic domains in panels (A)-(C) are shown in green ribbons, cyclins in cyan ribbons. (D) The superposition of the catalytic domains from the CDK5 structures (blue ribbons) and CDK9 structures (green ribbons).

Multiple crystal structures of CDK5-p25 [44], CDK6/V-cyclin [47], and CDK9-cyclin T complexes [48–50] were used in DMD simulations to characterize conformational ensembles of CDK proteins (Fig 1). The crystallographic conformations of CDK complexes featured important differences in the binding interfaces and position of the binding partners. The binding interfaces in the CDK5-p25 complex (Fig 1A) and CDK6/V-cyclin complex (Fig 1B) are similar and extensive, where functional regions from both lobes are engaged in the intermolecular contacts. The crystal structures of CDK5-p25 complexes showed a considerable similarity of the kinase domain conformation with only minor deviations in the terminal regions (Fig 1A). A slightly greater variability was seen in the crystal structures of CDK6-V-cyclin complexes, where major differences were localized in the N-terminal loop regions (Fig 1B). A significantly smaller binding interface is present in the crystal structures of CDK9-cyclin T that displayed a more significant conformational variability of the kinase domain (Fig 1C). These differences are spread across the catalytic domain and are more prominent in the N-lobe and the extended C-terminal tail that is unique to this transcriptional kinase. The alignment of multiple crystal structures for CDK5 and CDK9 can illustrate several conformational differences (Fig 1D). In particular, CDK9 structures showed appreciable displacements of the αC-helix position along with some variability in the β3-αC loop and the G-loop regions.

**Fig 2:**
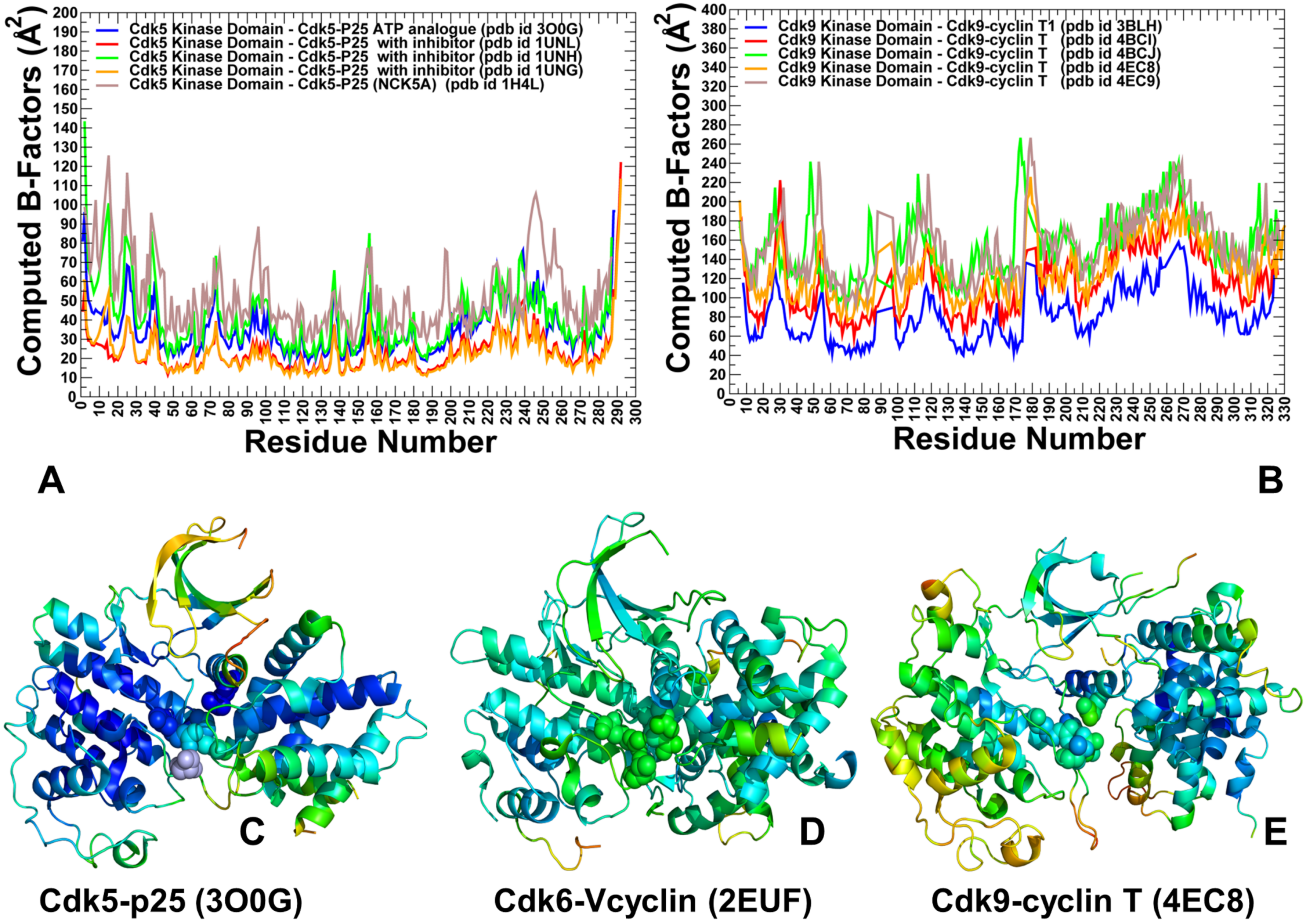
Conformational dynamics profiles of the CDK5-p25, CDK6/V-cyclin and CDK9-cyclin T complexes. (A) The computed B-factors obtained from simulations of the CDK5-p25 complexes (pdb id 14H4L, 1UNG, 1UNH, 1UNL, 3O0G). (B) The computed B-factors obtained from simulations of the CDK9-cyclin T complexes (pdb id 3BLH, 4BCI, 4BCJ, 4EC8, 4EC9). Structural mapping of conformational mobility profiles in the CDK5-p25 complex (pdb is 3O0G) (panel C), CDK6-Vcyclin complex (pdb id 2EUF) (panel D), and CDK9-p25 complex (pdb id 4EC8) (panel E). The color gradient from blue to red indicates the decreasing structural rigidity of the protein residues. The conserved functional residues R50, R125, R149 and S159 in CDK5 are shown in spheres colored according to conformational mobility (C). A similar group of CDK6 residues (R60, R144, R168, and T177) is highlighted in spheres in (D). Conformational mobility of a coordinating triad (R65, R148, and R172) and phosphorylation site pT186 site in CDK9 is depicted by mobility-colored spheres in (E).

Using DMD approach, we simulated multiple crystal structures of CDK5, CDK6 and CDK9 proteins and explored conformational landscapes of the unbound and bound CDK states (Fig 2). The obtained coarse-grained conformational ensembles of CDK structures were subsequently subjected to all-atom reconstruction using PULCHRA method [102] and CG2AA tool [103] that derived atomistic structures from simulation trajectories. The all-atom conformations were additionally optimized using the 3Drefine method [104,105] that utilizes atomic-level energy minimization with a composite physics and knowledge-based force fields. We analyzed protein flexibility by computing B-factors from simulations of the CDK crystal structures. The reported B-factors represent the average values obtained from multiple simulations runs (Fig 2A and 2B). A comparative analysis of conformational dynamics profiles in the CDK5 (Fig 2A) and CDK9 structures (Fig 2B) showed the increased values of B-factors in the CDK9 structures as compared to smaller thermal fluctuations in CDK5. Notably, the globally enhanced mobility of the CDK9 structures was not uniformly distributed, as major differences were localized in the G-loop, β3-αC loop, β4-β5 sheet, near the inter-lobe regions and in the C-terminal (Fig 2B). Structural mapping of the conformational mobility profiles highlighted a progressively increased mobility among CDKs, where CDK5 (nonclient) showed a considerable stability of the catalytic core (Fig 2C), CDK6 (weak client) displayed a moderately enhanced mobility (Fig 2D), and CDK9 (strong client) revealed an elevated mobility that was widely spread in the catalytic core (Fig 2E). In Cdk5, the β3-αC loop (residues 37-LDDDDE-42) showed relatively minor variations (Fig 2A and 2C). On the other hand, the longer β3-αC loop in CDK9 structures (50-VLMENEKEGF-59) could experience the greater flexibility (Fig 2B and 2E). As a result, steric constraints on the adjacent αC-helix can be partly removed in a highly dynamic N-lobe of CDK9 structures and allow for positional fluctuations between an active ‘αC-in’ conformation and intermediate positions. The detected dynamic changes can weaken functionally important coupling between the αC-helix and phosphorylation site in the T-loop, which may compromise structural stability of the active CDK9 state. Of particular interest were differences in the differential stabilization of the kinase lobes for CDK5 (nonclient) and CDK9 (strong client) structures. While the N-lobe regions in CDK5 structures experienced only minor fluctuations, the dynamics of the N-lobe in the CDK9 structures revealed considerably greater variations, particularly in the β3-αC loop and the regulatory αC-helix.

We also monitored conformational mobility of critical residues involved in the formation of active conformations in studied CDK structures. CDK5 does not require phosphorylation despite presence of S159 site at a position which is equivalent to the phosphorylation site in CDK2. In the CDK5-p25 complex, the conserved arginine residues (R50, R125 and R149) remain stable and come close together to form an interaction cluster with S159 in the T-loop, contributing to stabilization of the active conformation (Fig 2C). In the CDK6-Vcyclin structure, the phosphorylation site T177 can be coordinated by the three arginine resides R60 (αC-helix), R144 (HRD motif), and R168 (T-loop) that adopt similar conformations as in the phosphorylated CDK2 (Fig 2D). Although the extensive CDK6-Vcyclin interface can promote stabilization of the active kinase conformation, the intrinsic dynamics of the kinase domain can partly offset the stabilizing effect of the bound cyclin. Structural map of conformational mobility profiles illustrated these observations, showing stability of the CDK5 catalytic domain and rigidification of the binding interface (Fig 2C), whereas the T-loop regions maintained an appreciable degree of mobility in CDK6 (Fig 2D). In CDK9 structures, the open conformation of the T-loop is similar to that of CDK5 and CDK6 proteins, but the dynamic environment of the phosphorylation site (pT186) in CDK9 is quite different (Fig 2E). Due to enhanced flexibility and positional variations of the αC-helix, pT186 site can be properly coordinated only by R148 and R172, but lacks sustainable contacts with R65 from the αC-helix. As a result, functional regions in CDK9 are highly dynamic, leading to the reduced inter-lobe cooperativity and less robust activation. To summarize, conformational dynamics profiles revealed significant differences between structurally similar CDK proteins, particularly revealing that the elevated mobility of CDK9 client that can be contrasted to structural rigidity of CDK5 nonclient. These findings indicated that differences in chaperone dependencies among these CDK proteins may arise from their specific dynamic signatures that are manifested by differential stabilization of the kinase lobes.

To highlight the interplay between evolution and stability, we probed a relationship between conformational dynamics and sequence conservation in the CDK family. Using mutual information (MI) and coevolutionary analysis in the framework of MISTIC approach [106,107] we evaluated the Kullback-Leibler (KL) conservation score and coevolutionary relationships between position pairs in the kinase family. The sequence analysis identified a number of highly conserved residues shared by CDK proteins, stressing a strong correspondence between sequence conservation and structural stability (S2 Fig). Interestingly, a single most conserved residue is HRD-histidine, serving as a critical integrated center of the kinase core that links catalytic, regulatory and substrate-binding regions [8–13]. Structural analysis of the HxD motifs in multiple crystal structures of protein kinases showed a high degree of structural conservation of this residue in the activated protein kinases [14,15]. By mapping highly conserved residues on the crystal structures of CDK proteins, we illustrated a relationship between sequence conservation and structural stability of key functional regions. Among highly conserved sites in CDK5 are catalytic salt bridge pair K33, E51; functional residues D126 (HRD motif), K128, N131, D144 (DFG motif), F145 (DFG motif, R-spine), Y167, P170, D184 (R-spine), W186 (S2 Fig). Notably, these evolutionary conserved positions are also structurally rigid in CDK proteins. A number of other conserved residues are localized in the ATP binding site, the catalytic loop, and the substrate binding site. In general, the evolutionary conserved regions display a high degree of structural stability and often strategically positioned in the catalytic domain to mediate allosteric interactions and invariant functions of CDK proteins. This analysis suggested that divergences in the regulatory mechanisms and chaperone dependencies of CDK proteins may be associated with differences in global dynamics and allosteric coupling between structurally invariant and flexible regions involved in activation transitions.

**Fig 3:**
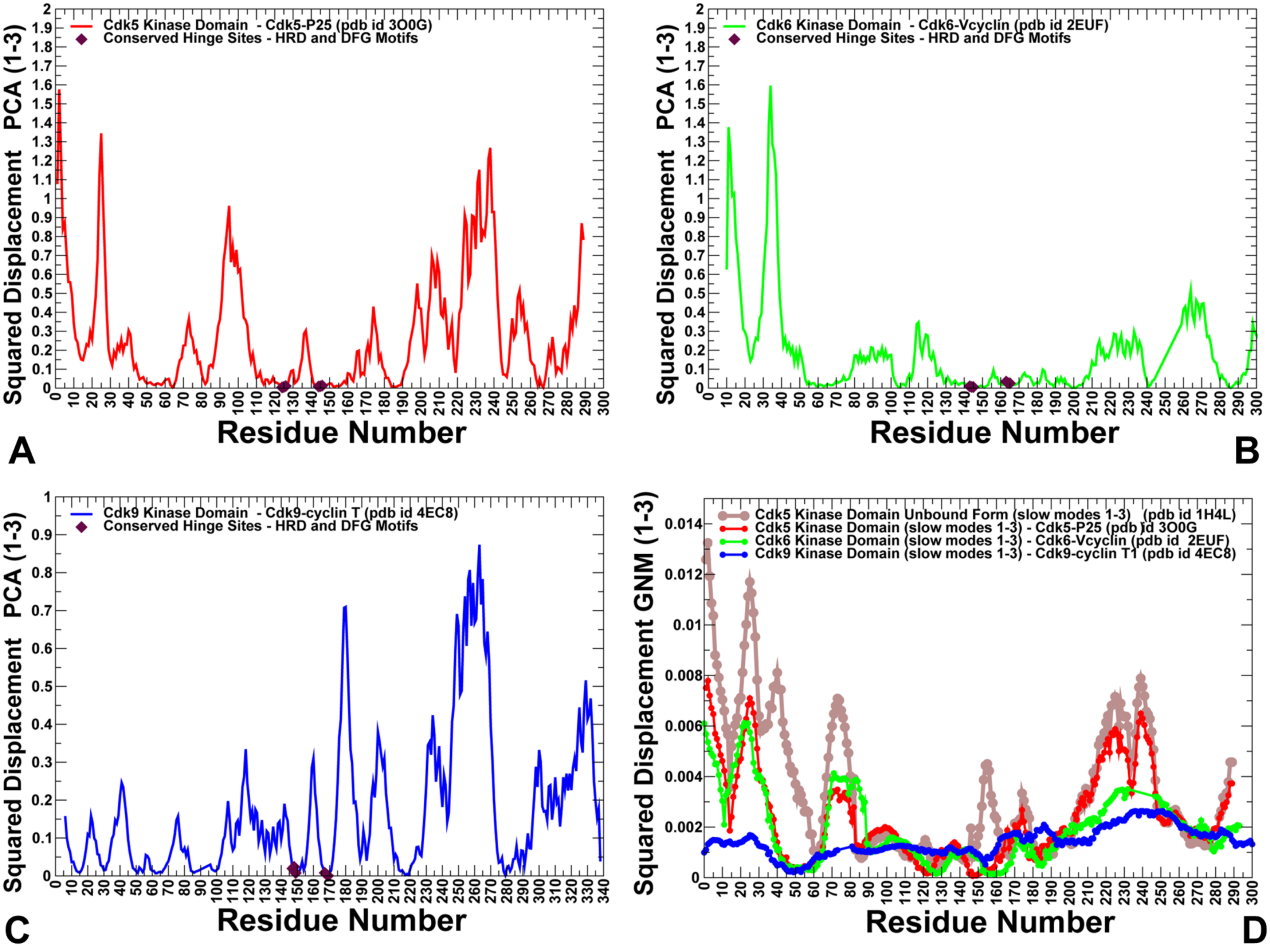
The essential dynamics profiles of the CDK5-p25, CDK6/V-cyclin and CDK9-cyclin T complexes: A comparison of PCA and GNM computations. A comparative analysis of the collective dynamics profiles in the CDK proteins. The normalized squared displacement of kinase domain residues averaged over first three PCA components for CDK5 (pdb id 3O0G) (in red lines); for CDK6 (pdb id 2EUF) (in green lines) (B); for CDK9 (pdb id 4EC8) (in blue lines). The positions of HRD and DFG motifs in these profiles are shown in filled maroon diamonds. (D) The GNM-derived essential mobility profiles in the space of the three slowest modes are shown for the unbound form of CDK5 (pdb id 1H4L) (in brown lines) and bound forms of the CDK5 catalytic domain from CDK5-p25 complex (pdb id 3O0G) (in red lines), CDK6 domain from CDK6-Vcyclin complex (pdb id 2EUF) (in green lines) and CDK9 domain from CDK9-cyclin T complex (pdb id 4EC8) (in blue lines).

To characterize differences in functional motions and collective dynamics of CDK proteins, we explored two complementary approaches: Principal Component Analysis (PCA) [108,109] and Elastic Network Modeling (ENM) [78,79]. In this analysis, PCA was used to extract principal components from DMD trajectories. We then compared the resulting principal components to the slow modes obtained from ENM calculations on the crystal structures. These approaches typically produce similar results and can provide robust assessment of functional dynamics for protein systems [110,111]. PCA of molecular dynamics (MD) trajectories using the heavy atoms representation of protein systems can arguably provide a more adequate description of slow modes of motion and yield a more accurate view of collective dynamics [112]. By using the reconstructed all-atom conformations derived from DMD trajectories, we adopted this protein representation in conducting PCA modeling of the CDK5, CDK6 and CDK9 structures. We found that the first three lowest PCA modes typically accounted for ~ 80–85% of atomic fluctuations in each trajectory. For all studied kinase structures, the first principal mode typically corresponded to the opening and closing movements of the kinase lobes with respect to each other. The second principal mode describes a shear motion between the N-terminal and C-terminal lobes, whereas the third principal mode corresponds to opposing movements of the C-terminal and N-terminal tails. The observed pattern of principal motions is conserved among protein kinase folds [113]. We computed the normalized squared displacements averaged over the first three principal components for representative CDK5, CDK6 and CDK9 structures (Fig 3A–3C). The local minima along these principal components usually refer to key functional sites serving as global hinge centers that control cooperative movements of subdomains, whereas peaks point to the most flexible regions, often involved in binding and substrate recognition [114]. We found that the αC-helix region, the catalytic HRD motif and the regulatory DFG moiety corresponded to conserved hinge sites that are shared by all CDK structures (Fig 3A–3C). Evolutionary and structural conservation of these regions may have contributed to their critical role in kinase activity and regulation of collective motions. In the CDK5-p25 and CDK6-Vcyclin complexes, binding activators can induce the expansion of the hinge cluster in the αC-helix (residues 43–57 in CDK5) and promote formation of an additional hinge center near the phosphorylation site of the T-loop (Fig 3A and 3B). In particular, the 144-DFG-146 hinge center in the CDK5-p25 complex expanded and included a group of adjacent T-loop residues (Fig 3A). An important distinction of the CDK5 activation mechanism is that the active T-loop conformation is not stabilized by phosphorylation, but rather by extensive interactions of S159 with the neighboring residues (R125, Y179) and binding activator. In the CDK5 complex, the expanded hinge site is centered on I153 and S159 residues that are immobilized during functional motions. In contrast, the hinge centers in the CDK9 complex are primarily localized in the αC-helix and HRD/DFG regions (Fig 3C).

To complement PCA results, we also explored ENM analysis of collective movements by using crystal structures of the studied CDK proteins (Fig 3D). Using Gaussian Network Model (GNM), the slow mode profiles along the three lowest frequency modes were computed for the unbound and bound forms of CDK proteins. A considerable conservation of the essential profiles was observed for CDK5 and CDK6 structures. We also found that PCA and GNM approaches yielded qualitatively similar results by predicting the same hinge site positons in the αC-helix region and near the HRD/DFG motifs. Our findings confirmed that these evolutionary and structurally conserved regions may be intrinsically predisposed to serve as regulatory centers of collective dynamics. The emergence of expanded hinge centers that are broadly distributed along the CDK5-p25 binding interface is also consistent with PCA results. This can ensure cooperativity of inter-lobe motions and contribute to the formation of a large allosteric interaction network in the CDK5 structures (Fig 3A and 3D). In some contrast, the local minima corresponding to hinge centers in CDK9 structures are narrow and localized (Fig 3C and 3D), allowing for larger movements of the T-loop and C-lobe around small CDK9-cyclin T interface.

**Fig 4:**
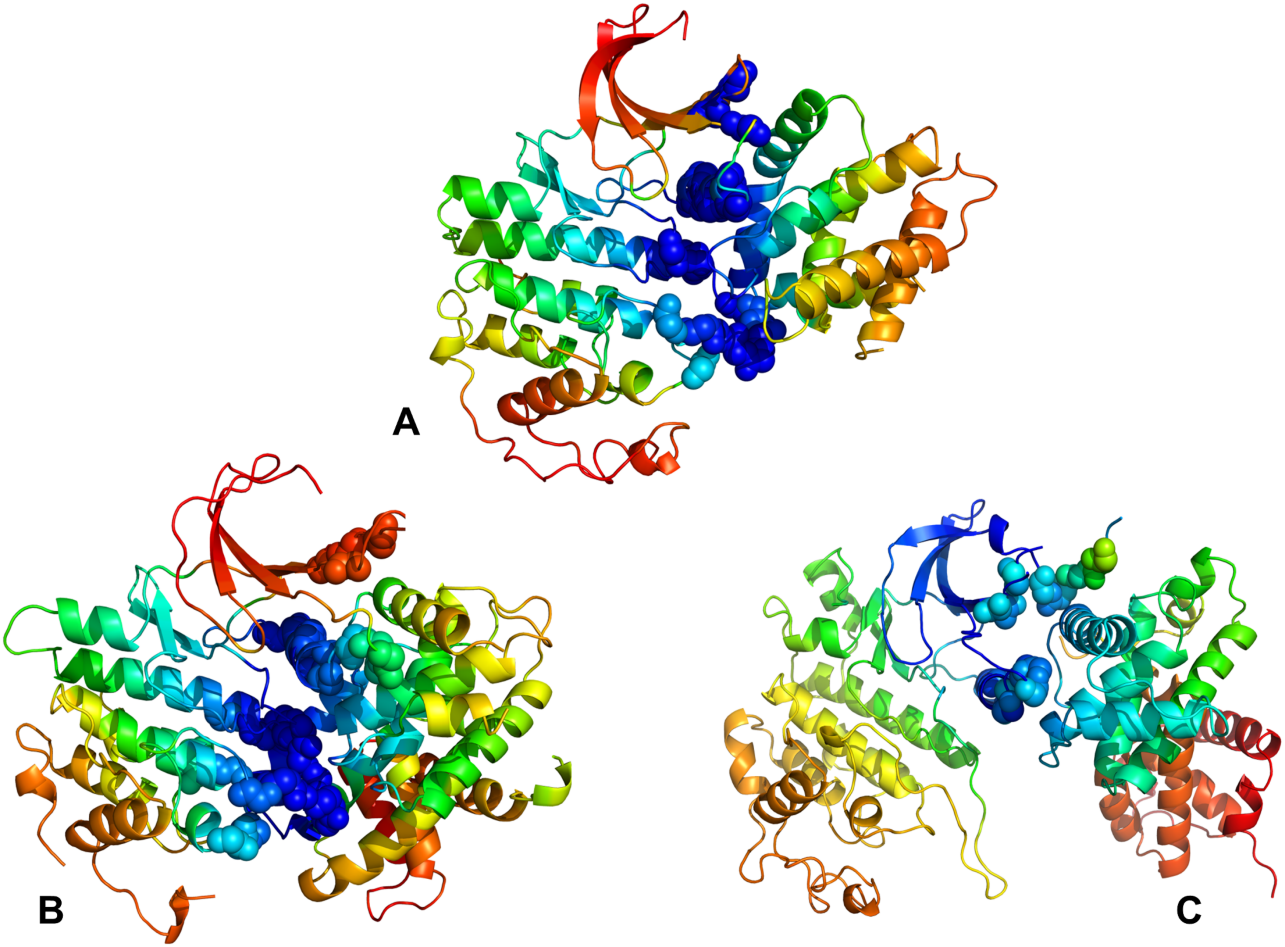
Structural mapping of collective dynamics profiles for the CDK5-p25, CDK6/V-cyclin and CDK9-cyclin T complexes. Structural mapping of the GNM-based collective dynamics profiles driven by the slowest three modes is shown for the CDK5-p25 complex (pdb id 3O0G) (A), CDK6-Vcyclin complex (pdb id 2EUF) (B) and CDK9-cyclin T complex (pdb id 4EC8) (C). The color gradient from blue to red indicates the decreasing structural stability (or increasing conformational mobility) of protein residues. The binding interface residues are shown as spheres colored according to their mobility.

Structural mapping of the GNM-derived essential mobility profiles highlighted positions of the hinge sites and their overlap with the binding interface residues (Fig 4). An extensive binding interface in the CDK5-p25 complex is fully immobilized in the global modes, with four distinct hinge points located in both kinase lobes (Fig 4A). At the same time, the interfacial regions in the N-lobe of CDK6 could become mobile during collective motions and cause partial opening of the intermolecular interface in the CDK6-Vcyclin complex (Fig 4B). Despite similar shapes of the slow modes, the distribution of hinge centers is partially altered in CDK9, which can promote larger displacements of the kinase domain (Fig 4C). This pattern of collective movements may weaken synchronization of the inter-lobe motions and modulate activation mechanism in CDK9 [115]. The monomeric form of the CDK domain is intrinsically predisposed to undergo inter-lobe movements but needs activation-promoting interactions with cyclins to facilitate reorganization of the T-loop and stabilize the catalytically competent state. In this mechanism [116], activators could shift thermodynamic preferences of the kinase ensemble towards active-like states and then induce reorganization of the T-loop to complete activation process. Our results suggested that partial redistribution of hinge centers in CDK9 structures may compromise cooperative movements of the αC-helix, T-loop and the inter-lobe regions. These factors may play role in driving kinase propensities for chaperone binding as the elevated dynamics of CDK9 domain and imbalances in cooperative collective motions can create favorable conditions for chaperone intervention.

### Alanine scanning and mutational sensitivity analysis identify energetic hotspots and quantify role of functional regions in stabilization of the kinase domain

**Fig 5:**
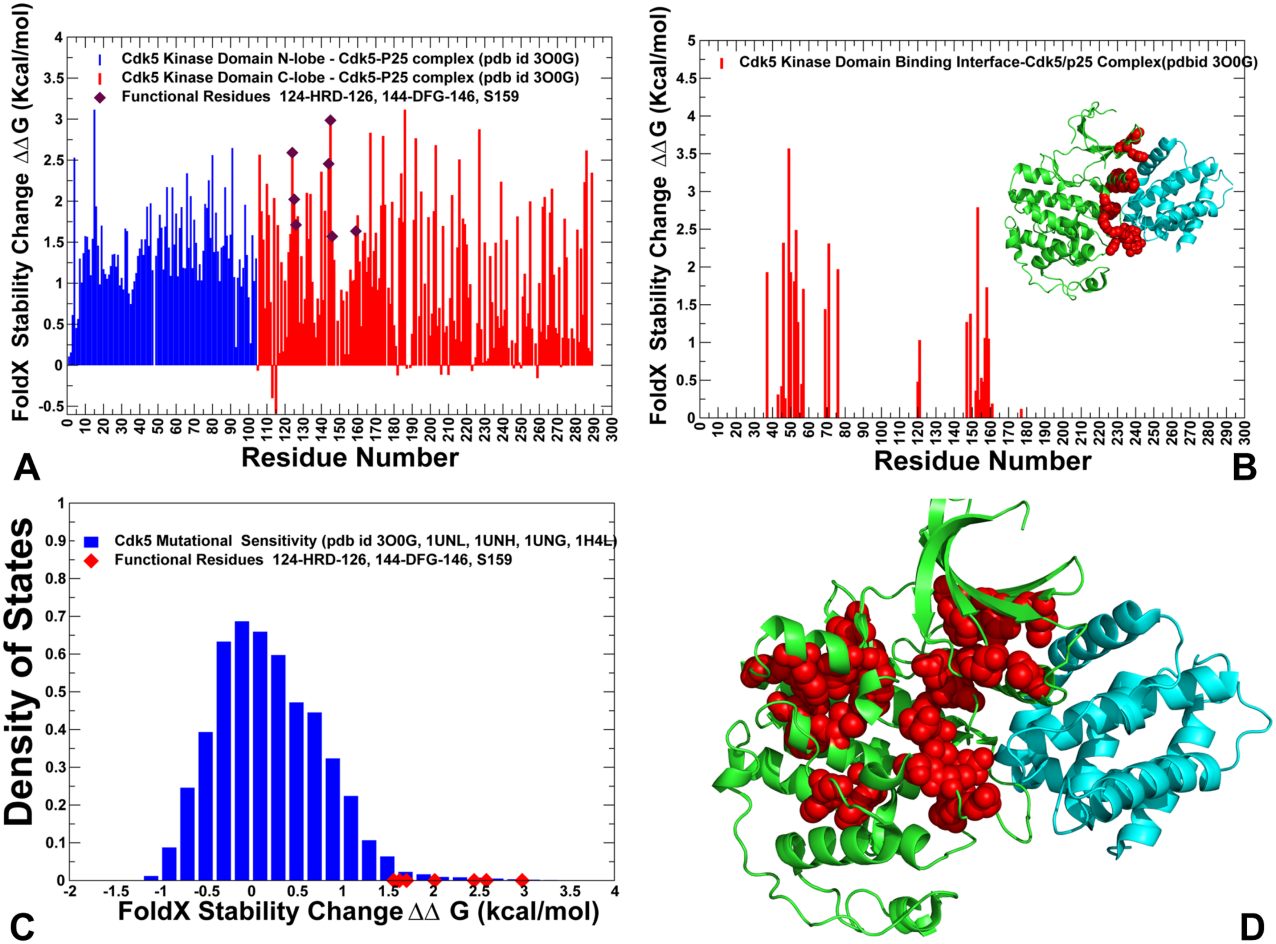
Protein stability analysis and mutational sensitivity profiles of the CDK5 structures. (A) The protein stability changes ΔΔG for the CDK5 catalytic domain residues are computed using a systematic alanine scanning of the protein residues to alanine and computing the effect of each mutation on protein stability with the FoldX approach. If the free energy change between a mutant and the WT proteins ΔΔG = ΔG (MT)-ΔG (WT) > 0, the mutation is considered to be destabilizing, and when ΔΔG <0 the mutation is stabilizing. The ΔΔG stability changes for the N-lobe residues are shown in blue bars and ΔΔG values for the C-lobe residues are shown in red bars. The positions of functional residues HRD, DFG and S159 are highlighted by filled maroon diamonds. The standard errors of protein stability changes, which are the standard deviation of the mean values, were ~ 0.1–0.2 kcal/mol. (B) The protein stability changes ΔΔG for the binding interface residues of the CDK5-p25 complex. The crystal structure of CDK5-p25 complex is inserted into graph and shown in ribbons (CDK5 is in green and p25 in cyan ribbons). The binding interface residues are highlighted in red spheres. (C) Mutational sensitivity analysis of CDK5 structures. The density distribution of ΔΔG values obtained from systematic mutations of protein residues in conformational ensembles of CDK5 structures (pdb id 3O0G, 1UNL, 1UNH, 1UNG, 1H4L). The ΔΔG values for the functional residues H124 (HRD), F145 (DFG) and S159 occupy the distribution tail and are highlighted by filled red diamonds. (D) Structural map of the energetic hotspots that produce a significant destabilization effect (ΔΔG > 1.5–2.0 kcal/mol) in the CDK5-p25 complex. The hotspot residues are shown in red spheres. CDK5 catalytic domain is in green ribbons, p25 is in cyan ribbons.

To determine differences in the energetics of CDK5 (nonclient) and CDK9 (client) structures and identify energetic hotspots, we conducted alanine scanning along with a mutational sensitivity analysis of protein residues in these structures. In the alanine scanning, we employed the FoldX force field method [117] implemented in the YASARA molecular graphics suite [118] and residues whose alanine mutations caused a significant destabilization effect (ΔΔG > 2.0 kcal/mol) were considered as energetic hot spots of thermodynamic stability. Statistically significant average ΔΔG values were obtained from 100 independent samples of conformational ensembles for each studied structure [119]. In this protocol, each sample consisted of 1,000 conformations randomly extracted from the conformational ensemble. The energetic profiles indicated that the active conformation of CDK5 was more stable, since alanine substitutions produced larger destabilizing changes (Fig 5A). Importantly, the average stability changes of the N-lobe and C-lobe residues in CDK5 were relatively similar. We found that strong destabilizing effects can be caused by mutations of Y15, L55, L66, L78, F80, H124, R125, L140, R149, I153, Y158, V163, Y167, F174, Y179, and D184 residues (Fig 5A). Of special interest was the emergence of the R-spine, HRD, and DFG residues as energetic hotspots of CDK5 stability. Significant destabilizing effect (ΔΔG > 2.0 kcal/mol) was caused by alanine mutations of the R-spine residues L55 in the regulatory αC-helix, L78 of the β4-strand (N-lobe), H124 (HRD motif), F145 (DFG motif), and D184 of αF-helix (C-lobe). Notably, F145A mutation in the DFG motif can have the most devastating effects on kinase stability and activity. This analysis confirmed an important role of the HRD and DFG motifs for the maintenance of the catalytically competent state (Fig 5A). The integrating position of this residue in the catalytic core, coupled with the role of the DFG motif in regulating activating transitions, may explain why drastic modifications of this residue can impair both stability of the catalytic domain and abolish kinase activity. These results supported the notion that single point mutations in the HRD and DFG motifs can significantly perturb energetics of the residue interaction network, indicating that the organization of these residues in the catalytic core of CDKs is relatively compact and inflexible. These results are consistent with mutational analysis of the DFG motif for p38 MAP kinase [120] and ABL kinase [121]. Another energetic hotspot corresponds to the H124 residue of the catalytic HRD motif (Fig 5A). In this case, H124A mutation resulted in the loss of hydrogen bond interactions with the aspartates of the HRD and DFG motifs, thus compromising coupling of the catalytic loop and the A-loop. The weakened hydrophobic interactions between the H124A and the DFG phenylalanine can lower stability of the R-spine and contribute to the in the formation of the weakened R-spine. Our findings agreed with the experiments showing that both of the hydrophobicity and hydrophilicity of the side chain of the HRD-histidine are important for full kinase activation [14,122]. Somewhat unexpectedly, the largest destabilization effect was observed for Y15A mutation in the N-lobe, suggesting that this position can be important for kinase stability and activity. These results appeared to be consistent with the recent experimental studies showing that Y15E, Y15F and Y15A can compromise stability of the N-lobe and severely impair kinase activity, though CDK5 can still bind to p35 activator [123].

Among energetic hotspots of CDK5 were also several other important residues including R125 (HRD motif), R149 and Y179 (T-loop). In CDK5, the conserved and stable arginine residues (R125 and R149) form an interaction cluster with Y179 and S159 in the T-loop (Fig 5A and 5D). The intermolecular interactions with the carbonyl oxygens of G238 and N239 on the p25 complete this important interaction cluster that stabilizes the T-loop and active conformation of CDK5. We noticed that S159 residue appeared to be more forgiving to alanine modifications (Fig 5A). This is consistent with the experimental data showing that S159A mutation cannot significantly alter the stability and activity of the CDK5-p25 complex reconstituted from recombinant proteins [124,125]. The biochemical studies suggested that although S159E and S159T may compromise CDK5 binding with p25 and p35 activators, these mutations have only moderate effect on stability of the kinase fold [44,124,125]. Through alanine scanning, we also estimated contribution of the binding interface regions to stabilization of the CDK5-p25 complex. Our analysis showed strong stabilizing contributions of the interfacial residues from the αC-helix (L49, C53, L54, E57) (Fig 5B). Another section of the binding interface that is important for stability corresponded to coordinating site R149, I153 (next to the DFG motif), C157 and Y158 residues. This analysis highlighted a critical contribution of the αC-helix interface to binding energetics.

We also performed a mutational sensitivity analysis of CDK5 by computing ΔΔG changes obtained from systematic substitutions of each protein residue (Fig 5C). The resulting distribution showed a shift in the ΔΔG values towards positive (destabilizing) contributions, also featuring a long tail that corresponds to the energetic hotspots. These findings reflected the overall stability of the active CDK5 structure, where the vast majority of substitutions can be destabilizing. Structural mapping of CDK5 residues that are important for stabilization of the active kinase highlighted tight packing and strong intramolecular interactions along the R-spine and C-spine (Fig 5D). Another revealing feature of energetically important residues in CDK5 is stability of the N-lobe residues near the regulatory αC-helix and rigidification of the inter-lobe regions that that are required for productive activation in the absence of phosphorylation.

**Fig 6:**
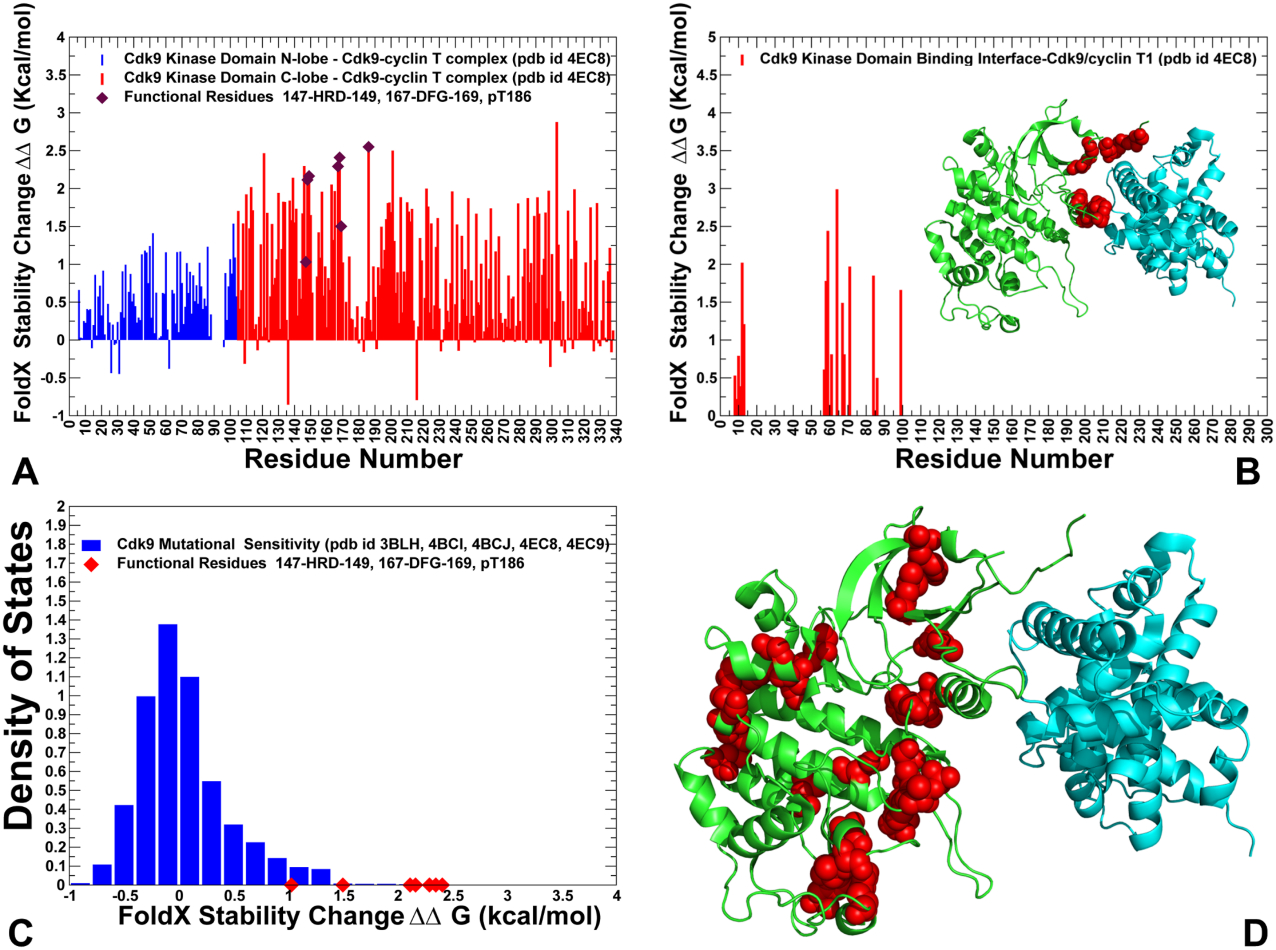
Protein stability analysis and mutational sensitivity profiles of the CDK9 structures. (A) The Protein stability changes ΔΔG for the CDK9 catalytic domain residues are computed using a systematic alanine scanning of the protein residues to alanine and computing the effect of each mutation on protein stability with the FoldX approach. The ΔΔG stability changes for the N-lobe residues are shown in blue bars and ΔΔG values for the C-lobe residues are shown in red bars. The positions of functional residues HRD, DFG and phosphorylation site pT186 are highlighted by filled maroon diamonds. The standard errors of protein stability changes, which are the standard deviation of the mean values, were ~ 0.2–0.35 kcal/mol. (B) The protein stability changes ΔΔG for the binding interface residues of the CDK9-cyclin T complex. The crystal structure of CDK9-cyclin T complex is inserted into graph and shown in ribbons (CDK9 is in green and cyclin T in cyan ribbons). The binding interface residues are highlighted in red spheres. (C) Mutational sensitivity analysis of CDK5 structures. The density distribution of ΔΔG values obtained from systematic mutations of protein residues in conformational ensembles of CDK5 structures (pdb id 3O0G, 1UNL, 1UNH, 1UNG, 1H4L). The ΔΔG values for the functional residues H147 (HRD), F168 (DFG) and pT186 occupy the distribution tail and are highlighted by filled red diamonds. (D) Structural map of the energetic hotspots that produce a significant destabilization effect (ΔΔG > 1.5–2.0 kcal/mol) in the CDK9-cyclin T complex. The hotspot residues are shown in red spheres. CDK9 catalytic domain is in green ribbons, cyclin T is in cyan ribbons.

A more significant disparity in the stability of the kinase lobes was seen in the CDK9 structures as mutations of the N-lobe residues resulted in small changes (Fig 6A). In particular, the β3-αC loop (50-VLMENEKEGF-59), the αC-helix (residues 60–72), and β4-β5 sheet (residues 82–104) could be relatively tolerant to substitutions, which may be determined by the higher mobility of these regions in CDK9 structures. The observed differential stabilization of the kinase lobes in CDK9 may compromise the fidelity of allosteric interactions and inter-lobe cooperativity, which is necessary to produce robust activating transitions. A similar pattern was observed in the energetic analysis of the binding interface residues for CDK9-cyclin T complex (Fig 6B), where mutations of only several hydrophobic residues (F59, L64) caused a significant destabilization. The mutational sensitivity analysis of CDK9 residues revealed a distribution with a characteristic peak corresponding to small positive ΔΔG values, featuring a shorter tail of energetic hotspots with larger destabilizing stability changes (Fig 6B). The emerging differences in the mutational sensitivity profiles of CDK5 and CDK9 structures reflected a considerable contrast between structural stability of CDK5 residues and elevated flexibility of the CDK9 domain, especially in the N-lobe. Structural mapping of the energetic hotspots in CDK9 pointed to a sparse and more fragmented organization of stable residues that tend to form small and isolated clusters. We argue that these energetic differences between CDK5 and CDK9 proteins may be linked with the corresponding divergences in their chaperone dependencies and strong client status of CDK9.

### Ensemble-based community analysis and modeling of communication pathways reveal depleted modularity of the allosteric interaction network in the client kinase

Using a graph-based representation of protein structures [94–96], we constructed and analyzed residue interaction networks in which dynamic contact maps of residue cross-correlations and coevolutionary residue dependencies define the strength of inter-residue edges between nodes [126–128]. In this model, allosteric communication pathways are determined by the ensemble of short inter-residue paths on a network graph that favor signal propagation through dynamically correlated and coevolutionary coupled nodes. We determined ensemble-based residue interaction networks in CDK proteins, where the strength of interaction edges was evaluated by averaging the measured correlated properties from multiple representative conformations in the ensemble. A global network parameter, residue centrality (betweenness) was used to identify mediating centers of allosteric interaction networks in CDK structures. The objective of this analysis was to test a hypothesis that organization and modularity of the interaction networks and mediating hotspots can be linked with the variations in structural stability and client status of CDK proteins.

**Fig 7:**
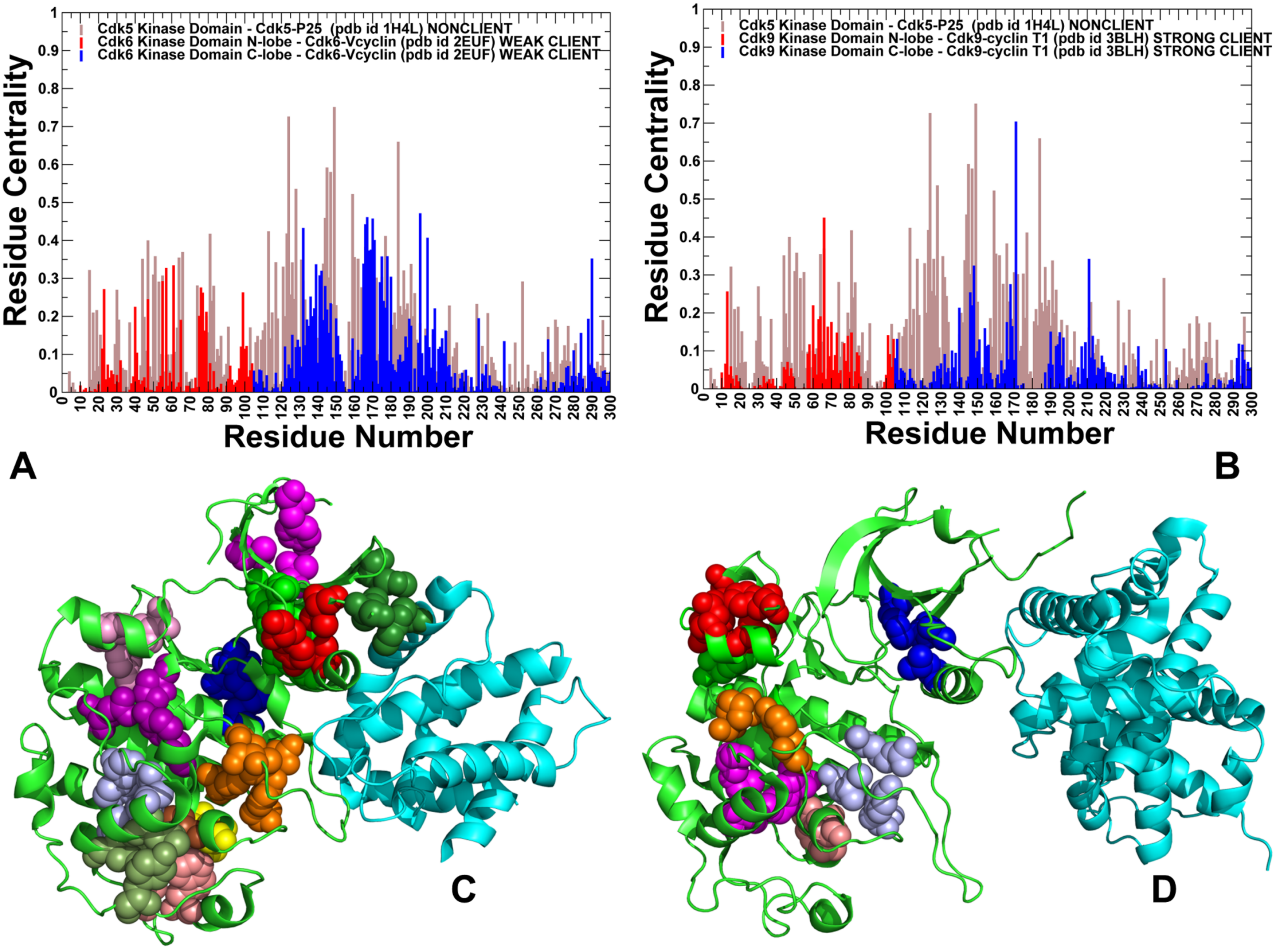
Analysis of the residue interaction networks and community maps in the CDK complexes. Residue-based centrality distributions of the CDK5-p25, CDK6/V-cyclin and CDK9-cyclin T complexes. The network profile of the CDK5-p25 residues (pdb id 3O0G) is shown in (A) and (B) in filled brown bars as a reference for comparison with the centrality profiles of the CDK6-Vcyclin structure (pdb id 2EUF) (panel A) and CDK9-cyclin T complex (pdb id 4EC8) (panel B). The N-lobe residues in the CDK6 and CDK9 centrality distributions are shown in blue bars and C-lobe residues are shown in red bars. The distributions are derived by averaging computations of network parameters over the conformational ensembles obtained from DMD simulations of the CDK5, CDK6, and CDK9 multiple crystal structures. Structural mapping of the local interaction communities in the CDK5-p25 complex (pdb id 3O0GT) (C) and CDK9-cyclin T complex (pdb id 4EC8) (D). Residue forming communities are show as spheres. Communities are shown in different colors. Structural maps of communities highlight differences in modularity of the residue interaction networks for CDK5 and CDK9 proteins.

In the network model, peaks in the centrality profiles can be attributed to mediating centers of allosteric interactions (Fig 7A and 7B). For convenience, the centrality profile of CDK5 was used as a reference in comparing differences with CDK6 *(*Fig 7A) and CDK9 proteins (Fig 7B). The distributions showed distinctly larger centrality values for nonclient CDK5, where broad peaks corresponded to the interfacial positions of the αC-helix (residues 43–55), catalytic HRD motif (residues 124–126) and at the T-loop region. The distribution featuring multiple and broadly distributed mediating clusters is characteristic of a large allosteric network in CDK5. Interestingly, the detected mediating centers of the interaction network overlapped with the position of hinge sites and binding interface hotspots. These observations indicated that key regulatory residues may coordinate multiple functions, including collective dynamics, propagation of allosteric interactions and binding with activating partners. Of particular interest was a single dominant peak located at the position of S159 in the T-loop of CDK5 (Fig 7A and 7B). This residue is conserved in all CDK5 orthologues and presence of a phosphate acceptor at this position was shown to be important for CDK5 regulation. Our findings singled out this position as an important mediating center of CDK5 regulation. These results are also consistent with functional studies of CDK5 regulation showing that phosphorylation in this position may negatively regulate CDK5 activity, while other mutations (S159E, S159T and S159A) may differentially affect CDK5-p25 binding and activity [124,125]. A comparison of the network profiles showed a progressively reduced centrality of the N-lobe regions in weak client CDK6 (Fig 7A) and a dramatic decrease in mediating propensities of CDK9 residues (Fig 7B). The reduced mediating capabilities of functional regions in CDK9 could lead to reorganization (rewiring or contraction) of the allosteric network that may compromise communication pathways and cooperativity in the kinase clients. To verify these conjectures, we performed community decomposition of the residue interaction networks in the CDK structures.

Community analysis revealed a dense web of stable interacting modules in CDK5 that allow for robust connectivity and coupling between functional regions (Fig 7C). Notably, local communities in the dynamic N-lobe allow for efficient allosteric coupling of G-loop, β3-αC loop, β4-β5 sheet, and α1-helix regions. The network organization in CDK5 featured a hierarchical nested structure with partially overlapping modules, which may ensure a proper balance of stability and functional adaptability in the system. Consistent with previous studies of community organization in protein kinases [129,130], we found that the regulatory spine residues in CDK5 (L57, L66, H124, F145, D184) were involved in the inter-modular bridges and enabled major lines of communications between local communities. In a sharp contrast, a sparse and fragmented network of local communities was observed in CDK9 structures (Fig 7D), where only few stable communities can be formed in the N-lobe regions. A dramatic transformation of the community map in the strong client CDK9 can be caused by the elevated dynamics, rendering a small and loose allosteric network. The observed reorganization showed clear signs of contraction in modularity of the CDK9 network, leading to reduction in allosteric coupling between functional regions. According to our results, the observed differences in the community organization are not caused by global rewiring of the network, but can rather arise from depleted modularity of the CDK9 network (Fig 7C and 7D). The observed dissipation of some local communities and inter-modular bridges in the CDK9 network was particularly evident in the dynamic N-lobe and near the inter-lobe regions. We suggested that the emerging ‘voids’ in the community map of CDK9 structures could affect the ensemble of short inter-residue paths and increase the average short path length in the network, thereby making allosteric communications in this client kinase less efficient.

**Fig 8:**
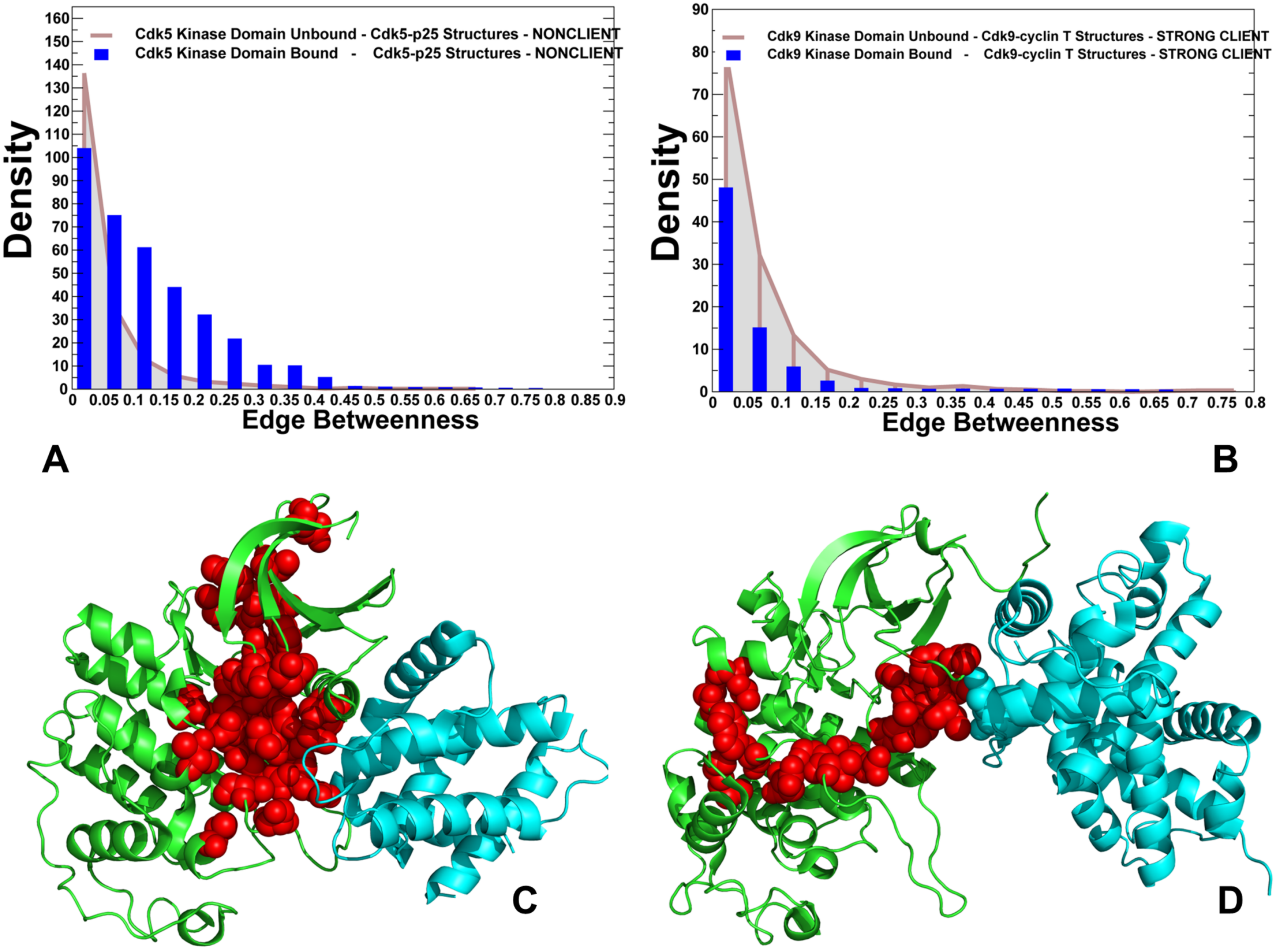
Network analysis of the ensembles of short paths and allosteric communications in the CDK structures. The edge centrality distributions for the unbound and bound forms of CDK5 structures (A) and CDK9 structures (B). The density of states distributions for the unbound kinase forms are shown in grey bars and for the bound kinase forms in blue bars. Structural mapping of high centrality edges in the CDK5-p25 complex (pdb id 3O0G) (panel C) and in the CDK9-cyclin T complex (pdb id 4EC8) (panel D). The kinase domains are shown in green ribbons and binding partners are shown in cyan ribbons. The residues forming high centrality edges are shown in red spheres. For clarity, high centrality connectors are presented only for the kinase domains.

To substantiate these arguments, we evaluated the ensemble of shortest inter-residue paths in CDK structures by computing the edge betweenness (centrality) for each pair of residues using the average values over conformational ensembles. This network parameter is defined as the number of shortest paths in the total ensemble that proceed through a given edge. The edges of high centrality values represent the inter-modular bridges that direct most of the communication traffic in the system. Accordingly, the removal or alteration of these bridges may affect allosteric communications between many pairs of residue nodes by changing the shortest inter-residue routes. Computation of the ensemble of short paths between any pair of residues was based on the community decomposition by the Girvan-Newmann algorithm [131–133]. This method utilizes the edge betweenness as a partitioning criterion and splits network into local communities via an iterative procedure, in which the edge with the highest centrality is removed from the network and the betweenness of the remaining edges is recalculated. We compared the edge centrality distributions in the unbound and bound forms of CDK5 structures (Fig 8A) and CDK9 structures (Fig 8B) that represent the opposite sides of the chaperone dependency spectrum. The distributions for the unbound CDK5 and CDK9 kinase domains were similar, featuring a sharp decline and long tail which are characteristic of small-world network organization [94]. In CDK5 complexes, the distribution showed a significant density for the inter-residue edges with medium-to-high centrality values (Fig 8A). Accordingly, allosteric communications in CDK5 may explore a broader ensemble of probable routes, allowing for efficient signaling between functional regions. In some contrast, the distribution for CDK9 structures revealed a dominant peak corresponding to low edge centrality values and a long dissipating tail of edges with higher betweenness (Fig 8B). This implies that allosteric pathways in CDK9 structures may preferentially proceed through a small number of critical bottlenecks.

We argue that this divergence of global network characteristics can be associated with underlying differences in chaperone dependencies of CDK5 and CDK9 proteins.

Structural mapping of high centrality edges exposed probable routes of allosteric communications and highlighted key differences between ensembles of short paths in the CDK structures (Fig 8C and 8D). We observed that functional regions in the CDK5 structures can be efficiently connected through several main communication routes (Fig 8C). The topography of high centrality edges in CDK5 revealed that regulatory regions from both kinase lobes are strategically positioned in this map, bridging the nucleotide binding site with the phosphorylation center and substrate binding site. The ensemble of communication routes connecting the ATP binding site and the T-loop can utilize a dense network of high centrality edges, where most critical global bridges (H124-R149, H124-D184, D126-L147, S159-V162) are formed by functional residues from the HRD motif and R-spine residues (Fig 8C). According to our analysis, these sites can serve as key allosteric hubs of signal transmission in CDK5 structures. Importantly, high centrality bridges in CDK5 were not isolated and could be surrounded by supported by neighboring residues with sufficient communication capabilities to ensure resilience of allosteric signaling. A radically different map of high centrality edges emerged for the CDK9 structures, featuring a fairly narrow funnel that connected the binding interface residues through the HRD motif and the C-lobe regions (Fig 8D). Several high centrality edges in the CDK9 map (I61-R65, R65-E66, and R65-I69) connected the αC-helix with the catalytic core. Another group of highly populated edges included R148-V189 and R148-V190 that engaged coordinating residue R148 involved in interactions with the phosphorylation site pT186 in the T-loop. These high centrality bridges represent key bottlenecks that are involved in propagating allosteric signals in CDK9. A low participation of the N-lobe residues in the main routes indicated that allosteric communication between kinase lobes may become less efficient due to longer paths connecting functional regions. These findings confirmed that depletion of community maps in CDK9 structures can affect the ensemble of short inter-residue paths by forcing communications via a narrow propagation route and increasing the average short path length in the network. This may render longer and less efficient inter-residue communications in CDK9 complexes. To summarize, community analysis and modeling of communication pathways in CDK structures suggested that differences in modularity of the allosteric interaction networks can be linked with chaperone dependencies and variations of the regulatory mechanisms.

In network terms, our results imply that mutations of key mediating centers from the HRD and DFG motifs may severely impair the efficiency of allosteric communications in CDK5 and CDK9 proteins. At the same time, the adverse effects of mutations in other positions could be potentially mitigated in CDK5 because of a large allosteric network and presence of alternative routes between the kinase lobes. To substantiate these arguments, we performed DMD simulations analyzing conformational ensembles and residue interaction networks for several representative CDK5 mutants F145A (DFG motif) and S159A. According to our hypothesis, the known severe effect of the F145A mutant on kinase stability and activity should manifest in global alterations of the residue interaction network and irreparable damage to allosteric communications in CDK5 structures. At the same time, we proposed that a detrimental effect of S159A mutant on the allosteric interaction network could be less dramatic and weaken allosteric signaling in CDK5 rather than completely abolishing activity. The central finding of this analysis was that CDK5-F145A mutation can induce significant global changes on the dynamics of the catalytic domain by increasing flexibility of the core regions that manifested in the drastically lowered centrality of the αC-helix (residues 43–55), catalytic HRD motif (residues 124–126), DFG motif (residues 144–146) and the T-loop as compared to the CDK5-WT protein (S3 Fig). Dynamic coupling between these functional regions is fundamental for kinase activity, and the observed reduction in their mediating capabilities can severely impair allosteric interactions between the kinase lobes that are required for productive activation.

Structural mapping of high centrality edges in the CDK5-F145A structure highlighted the observed changes in the distribution of major communication pathways. The network of inter-residue bridges becomes sparser and more fragmented, consisting of small isolated clusters (S3 Fig). According to our analysis, the F145A mutation could impair the dominant ensemble of short inter-residue pathways that use the HRD and DFG motifs to connect the nucleotide binding site and the αC-helix with the T-loop and CDK5-p25 binding interface. The alternative routes connecting the kinase lobes utilized other bridges (K128-Y167, N131-D144, K128-L132, K128-N131, V64-L66, and V64-E81) that included only a single R-spine residue (L66), as structural integrity of the R-spine was irreparably damaged by F145A mutation. The observed dislocation of communication hubs that diverted signaling routes away from functional regions can impede allosteric coupling and preclude activation driven by the assembly and stabilization of the hydrophobic R-spine. These results confirmed a central role of the DFG motif and the R-spine network in mediating allosteric interactions and activation mechanisms. At the same time, S159A mutation caused a moderate effect on residue centrality profile and distribution of high centrality edges (S3 Fig). Although we observed globally distributed changes in the residue centrality, the mediating capabilities of HRD and DFG motifs were not significantly affected in the CDK5-S159A mutant. Moreover the centrality of the αC-helix region (residues 43–55) even moderately increased, indicating that CDK5-S159 mutant would maintain allosteric coupling between functional regions required for activation. For this CDK5 mutant, the topography and density of communicating centers was mostly preserved and the key inter-residue bridges (H124-R149, H124-D184, and D126-L147) retained their hub status (S3 Fig). However, several important high centrality bridges (R125-Y179 and S159-Y179) were broken in CDK5-S159A mutant, leading to less efficient coupling between the catalytic core (HRD motif) and the T-loop. The preservation of a dense allosteric network in the CDK5-WT and CDK5-S159A proteins suggested that the adverse effect of S159 mutations on the allosteric interactions can be partly attenuated as the existence of many alternative routes would still ensure propagation of the activation signal. These results are consistent with functional studies showing that S159A mutation has a relatively moderate effect on p25 binding and kinase activity [44]. The network analysis of CDK5 mutants showed that although mutations of functional residues may often result in a dramatic loss of signaling activity, some of these changes could be tolerated in a broad network of mediating centers, where other residues may fulfill functional responsibilities in the altered interaction network.

### Perturbation response scanning and effector residue propensities link differences in allosteric communications with client status

To further substantiate the results of network analysis and quantify role of functional residues as mediators and propagators of dynamic fluctuations and allosteric interactions, we used the perturbation-response scanning (PRS) approach [134–136] that was integrated with the GNM formulation [137,38]. In this approach, a perturbation force is applied to the network, one residue at a time, and the response of the overall network is measured according to Hooke's law as a displacement vector **ΔR**(*i*) = **H**^**-1**^**F**^**(***i*)^ that is then translated into *N*×*N* PRS matrix, **S**_PRS_. In this matrix, the *ij*_th_ element evaluates the sensitivity of mode *i* to perturbation at position *j*. By using this approach as implemented in [137], we obtained the PRS maps where the row *i* describes the response of the residue *i* to perturbations in other sites (Fig 7). The average values computed over all elements of the PRS matrix in the corresponding row measure the ability of a given node to propagate perturbations to other nodes in the system. The respective residue profiles provide information about average mediating capabilities of a given residue (termed effector or influencer) in transmitting signals when subjected to a unit perturbation. According to the PRS model, the peaks in the effector profiles would correspond to sites that can best absorb and transfer the perturbations and dynamic fluctuations throughout the protein to all other residues, thus quantifying the role of a given residue as a potential mediating hotspot in the allosteric interaction network [138].

**Fig 9:**
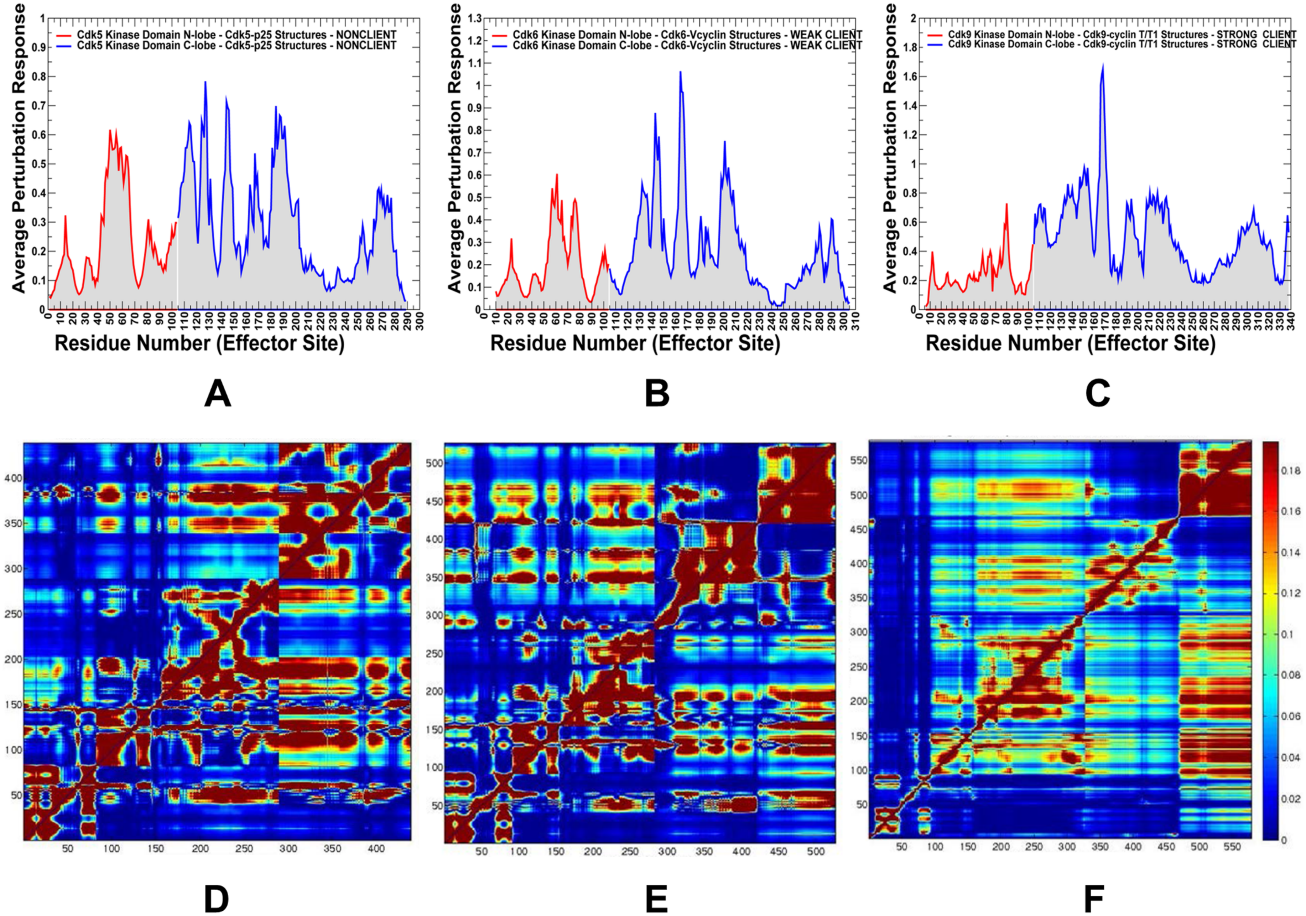
Perturbation-response scanning analysis and the effector residue profiles of the CDK structures. The residue-based effector profiles are shown for the CDK5-p25 complex (pdb id 3O0G) (panel A), CDK6-Vcyclin complex (pdb id 2EUF) (panel B), and CDK9-cyclin T complex (pdb id 4EC8) (panel C). These distributions show the average propensity of kinase residues to transmit perturbation. The effector value for each residue is computed as the average over all elements of the PRS matrix in the corresponding row. The distributions are annotated as follows: the effector values for the kinase N-lobe residues are in red lines, C-lobe residues are in blue lines. For clarity of presentation, the PRS profiles are shown only for the kinase domain. The PRS heat maps are shown for CDK5-p25 (D), CDK6-Vcyclin (E) and CDK9-cyclin T structures (F). These heat maps highlight the strength of the response of perturbations and color-coded from low preferences to act as effectors (in blue) to high propensities to act as effector (in red). The heat maps are shown for complete complexes and include contribution of the kinase domain and binding partner.

A comparison of the effector residue profiles showed a steady shift in the distributions from nonclient CDK5 (Fig 9A) to weak client CDK6 (Fig 9B) and strong client CDK9 (Fig 9C). The broadly distributed multiple peaks in CDK5 corresponded to regulatory residues from both lobes, including residues 50–64 (αC-β4/αC-helix region), residues 124–126 (HRD motif), residues 144–146 (DFG motif at the beginning of the T-loop) and residues 186–192 (integrating αF-helix in the C-lobe). These mediating centers coordinate allosteric interactions and synchronize collective dynamics between the nucleotide binding site, the regulatory αC-helix, the integrating αF-helix and the substrate binding site. The strategic location of these residues and their strong influence on cooperative fluctuations in the binding sites suggest an important role in establishing allosteric communication in the CDK5 complex. One of the central finding of the PRS profiling was a progressively diminished role of the N-lobe residues in the effector profiles for the weak client CDK6 and strong client CDK9 (Fig 9B and 9C). In the CDK6 profile, we noticed the lowered peaks in the N-lobe (E61 and V76 residues from the αC-helix region), with the major mediating centers H143 (HRD motif), F164 (DFG motif), and D201 (R-spine residue in the C-lobe). A further dissipation of the effector centers was seen in the CDK9 structures (Fig 9C), where the peaks in the αC-helix and HRD regions were lowered, and a single major effector center resided at the regulatory DFG region. The heat map of residue responses to perturbations highlighted a cooperative nature of the allosteric network in CDK5 (Fig 9D), as residues with the increasing effector propensities (in red) formed clusters and occupied key functional positions in both lobes. The emergence of multiple communication hotspots that mediate signaling in the CDK5 complex was also consistent with the centrality and pathway analyses, confirming a large allosteric network. The residue response heat map showed weakening in the mediating strength of CDK6 residues (Fig 9E), causing the reduced allosteric coupling of the active conformation. The heat map highlighted the depletion of mediating centers in the N-lobe of CDK9 structures along with the reduced effector propensities of the C-lobe residues (Fig 9F). These results provided additional evidence that propagation of dynamic fluctuations and efficiency of signal transmission in CDK9 may be reduced due to dislocation of mediating centers and a smaller allosteric interaction network.

**Fig 10:**
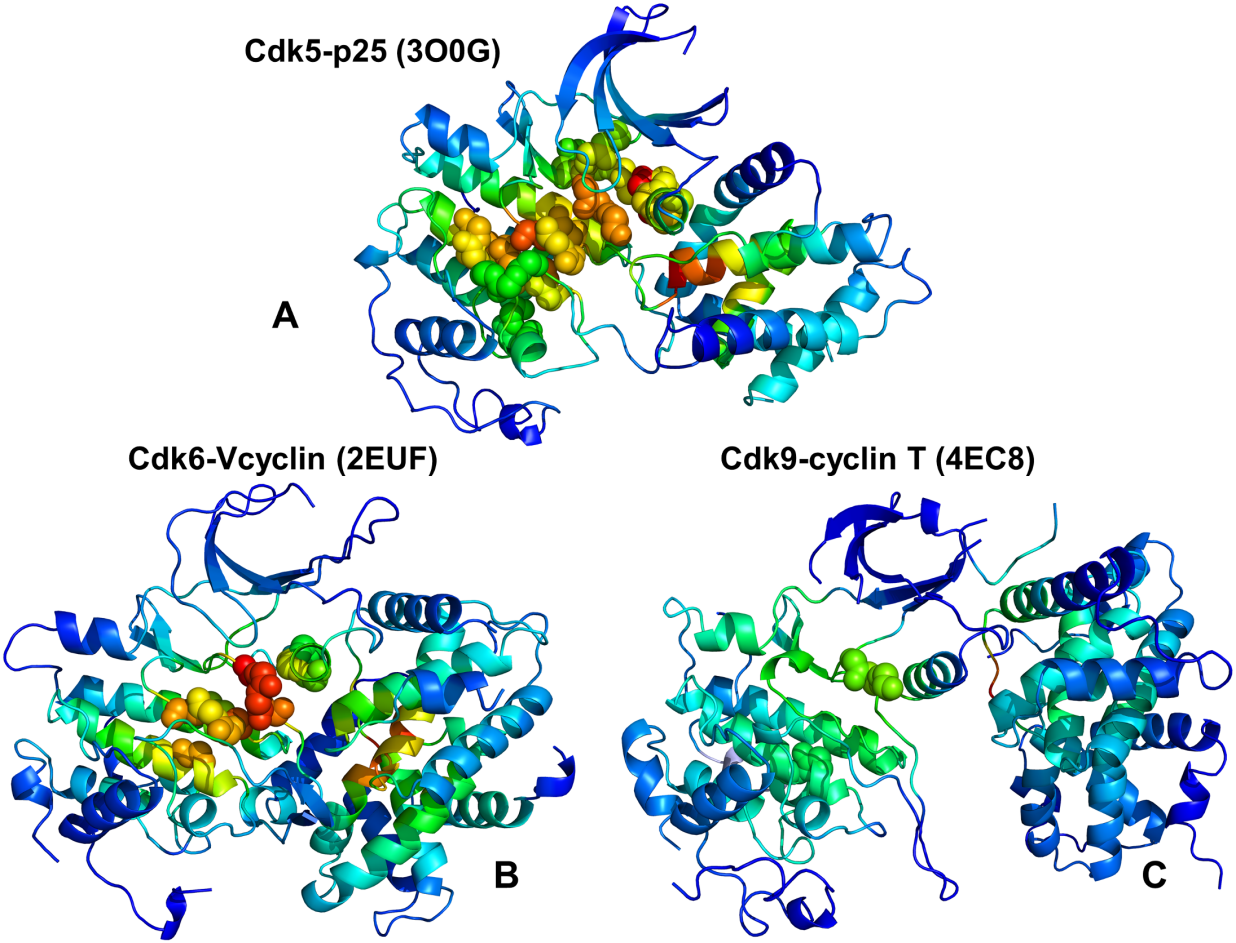
Structural maps of the effector propensities in the CDK structures. Structural mapping of the effector propensities is shown for the CDK5-p25 complex (pdb id 3O0G) (panel A), CDK6-Vcyclin complex (pdb id 2EUF) (panel B), and CDK9-cyclin T complex (pdb id 4EC8) (panel C). The structural maps are color-coded from low preferences to act as effectors (in blue) to high propensities to act as effectors (in red). The maps are shown for complete complexes and include contribution of the kinase domain and binding partner. The most influential effector sites in PRS profiles corresponding to the dominant peaks in the PRS effector profiles are shown in spheres that are colored according to the effector propensities. Structural maps highlight the greater number and the higher density of effector peaks (mediating clusters) in CDK5 (A), the decreasing density of effector clusters in CDK6 (B), and a small number of isolated effector centers in CDK9.

We also mapped the most influential effector sites (profiles peaks) onto the crystal structures of CDK proteins (Fig 10). This analysis illustrated differences in localization and connectivity of major mediating clusters. While major mediating centers (effector hotspots) in CDK5 structures are broadly distributed in the catalytic core (Fig 10A), the density of mediating clusters was partly reduced in CDK6 (Fig 10B) and significantly diminished in CDK9 structures (Fig 10C). According to these heat maps, both lobes in CDK5 structures can harbor effector hotspots and only several small segments of the kinase domain do not participate in the allosteric network (Fig 10A). These effector sites in CDK5 are situated in the regulatory regions and form dense clusters that enables efficient propagation of fluctuations in a large allosteric network. These observations confirmed the existence of a large allosteric network in CDK5 structures that may impede CDK5 recruitment to the Hsp90-Cdc37 system as the chaperone preferentially targets the intrinsically dynamic kinase folds with limited or impaired allosteric coupling. A proliferation of decoupled regions could be seen in the weak client CDK6 (Fig 10B) and especially apparent in the strong client CDK9 (Fig 10C). A small number of mostly isolated effector peaks (in the DFG motif) along with the increased density and size of decoupled regions in the N-lobe in CDK9 could imply a reduced cooperativity and a limited fragmented allosteric network in the client kinase (Fig 10C). These factors may play an important role in rendering CDK9 as a strong chaperone client since Hsp90-Cdc7 chaperone system that tends to recognize the unstable kinase states with reduced cooperativity and impaired allosteric interactions. Our results may help in providing structural rationale to the experimental evidence that the monomeric CDK9 can be highly susceptible to fast degradation and must form transient complexes with chaperones to facilitate association with the cyclin partners and formation of stable regulatory assemblies [139–141].

In summary, the perturbation response scanning identified regulatory hotspots that mediate allosteric interaction networks in the CDK structures. Importantly, the PRS results are fully consistent with community decomposition and pathway modeling analyses, showing that the intrinsic kinase dynamics can affect the distribution of mediating centers and network modularity, producing deviations in regulatory responses and chaperone dependencies. We also found that the effector sites in CDK structures correspond to high centrality residues and hotspots of allosteric communication pathways. The observed consistency in the prediction of allosteric mediators by different approaches and agreement with the experiments provided support to our computational predictions.

### Rigidity decomposition analysis and emulation of thermal unfolding relate differences in stability and allosteric cooperativity to chaperone dependencies of CDK proteins

Finally, we combined network modelling with rigidity-based decomposition analysis to emulate thermal unfolding and characterize distribution of rigidity and flexibility in the CDK proteins. The underlying hypothesis behind this approach is that imbalances in the distribution of rigid and flexible regions in the client kinase may weaken allosteric interactions and make the kinase fold susceptible to chaperone intervention and recruitment. We investigated how redistribution of flexibility and rigidity can modulate allosteric communications, cooperativity and chaperone dependencies of CDK proteins. Using FIRST approach [142–146] and the Python-based Constraint Network Analysis (CNA) interface [147,148] we performed network-based decomposition of CDK structures into rigid clusters and flexible connections. In the FIRST approach, thermal unfolding of protein structures was emulated by gradually removing non-covalent constraints from the constraint network and applying the pebble game algorithm to each of the resulting networks. This algorithm determines whether a bond is flexible or rigid and decomposes the constraint network into rigid clusters and flexible regions. A rigid cluster is a set of residue nodes that move together as a rigid body, whereas residues that are not a component of a rigid cluster are assigned to a flexible region. During unfolding, the weak constraints are removed first while stronger interactions are sustained longer, leading to progressive decomposition into rigid and flexible regions. We monitored the evolution of the ‘giant’ rigid cluster that disintegrates and breaks apart into a number of smaller rigid clusters during unfolding phase transition. During rigid cluster decomposition residues that break away from the giant rigid cluster near the transition point and become flexible are identified as ‘weak spots’ [149,150]. In thermal unfolding simulations, we incorporated conformational ensembles of CDK structures and used 1,000 representative samples from the trajectories to compute the frequencies for all residues to become weak spots at the unfolding transition point.

**Fig 11:**
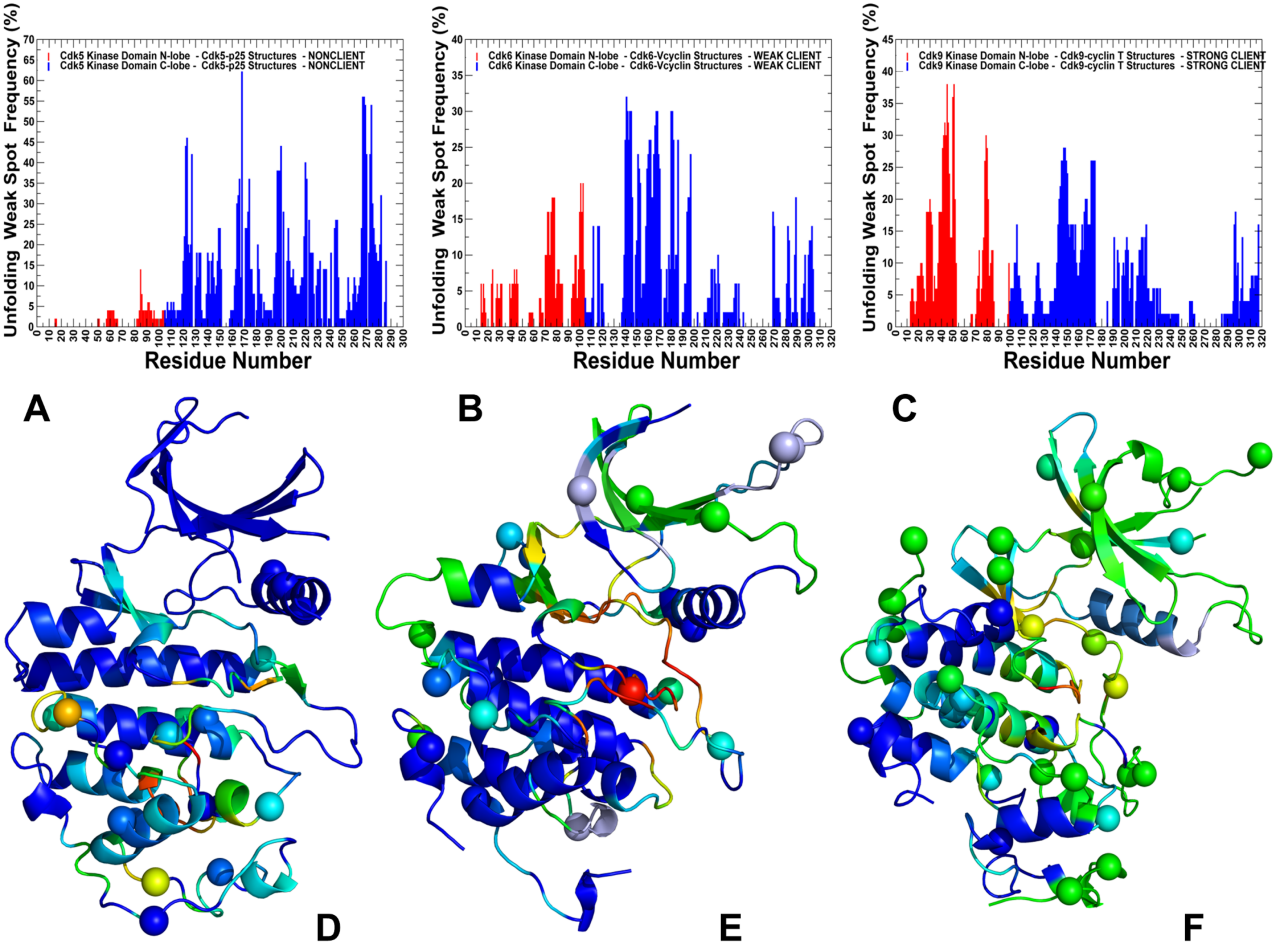
Rigidity analysis of thermal unfolding in the CDK structures. The frequency of unfolding nuclei (or weak spots) in the CDK5-p25 structure (A), CDK6-Vcyclin (B) and CDK9-cyclin T structure (C). The frequencies of the kinase domain residues are shown in colored bars, with the N-lobe residues in red bars and C-lobe in blue bars. Unfolding nuclei or weak spots are defined as residues that belong to the giant rigid cluster until the folding/unfolding transition point and break away from the giant rigid cluster immediately after transition during the network-based emulation of thermal unfolding. The localization of high frequency weak spot residues characterizes protein stability and rigidity/flexibility partition in the protein structure. The higher the frequency of a weak spot, the more probable unfolding begins from these residues. Structural mapping of predicted weak spots on structures of CDK5 kinase domain from CDK5-p25 complex (D), CDK6 kinase domain from CDK6-Vcyclin complex (E) and CDK9 domain from CDK9-cyclin T complex (F). Crystallographic conformations are colored using a color range from red (highest ranking weak spot) to blue (lowest ranking weak spot). The positions of pathogenic mutations in CDK structures are shown in spheres colored according to weak spot ranking.

The rigidity decomposition and structural mapping of weak spots showed clear differences between CDK proteins (Fig 11). In CDK5 structures, a large rigid cluster included the N-lobe residues, the inter-lobe region and the C-lobe core (Fig 11A). These stable regions act cooperatively during formation of a rigid phase. The departing flexible cluster at the transition point was relatively small as weak spots included only a segment of the T-loop and mobile residues in the C-terminal regions (Fig 11D). The network emulation of thermal unfolding showed that CDK5 structures are more rigid and are characterized by a large and cooperative allosteric network that connects functional regions from both lobes. A progressive increase in the number of flexible regions leaving a giant cluster during unfolding transition was seen in CDK6 (Fig 11B), where mobile clusters emerged in the N-lobe and included residues from the G-loop, β3-αC loop, β4-β5, and β6-β7 strands (Fig 11E). The β3-αC loop is believed to play an important functional role acting as a flexible ‘rheostat’ that modulates dynamics of the adjacent αC-helix and controls kinase activity [151]. The results pointed to a significant redistribution of rigid and flexible regions in the kinase clients as evidenced by gradual expansion of flexible regions in CDK6 and CDK9. These alterations in stability of the kinase regions were not uniform and differentially affected kinase lobes, where most of the flexible regions emerged in the N-lobe. Interestingly, the entire layered β-sheet structure of the N-lobe becomes more flexible in the CDK6 and CDK9 structures. In this context, it is worth noting that instability of the β-sheet regions is believed to be a common dynamic characteristic shared by many kinase clients of the Hsp90-Cdc37 chaperone [72]. The observed redistribution of rigidity in CDK9 client highlighted the proliferation of flexible clusters in the G-loop, β3-αC loop, and β4-β5 regions of the N-lobe (Fig 11D and 11F). These results allow an interesting interpretation of the first cryo—electron microscopy structure of the Hsp90-Cdc37-Cdk4 kinase complex [76] in which partially unfolded β3-αC loop and β4-β5 sheet are trapped by the interactions with Hsp90. Consistent with these experiments, network emulation of thermal unfolding showed that these segments would correspond to the weak spots of CDK9 client that can be targeted by the chaperone system during kinase recruitment.

Our findings are also consistent with several studies of protein kinases that suggested that dynamic and energetic polarization of the kinase domain lobes and conformational plasticity of the N-lobe in oncogenic kinases, such as EGFR and BRAF, may have favored selection of activation cancer mutations in flexible regions [152,153]. According to these studies, the distribution and balance of intrinsically rigid and flexible regions in protein kinases may dictate localization of activating mutations that could readily modulate conformational changes by targeting flexible regions without compromising structural stability of the kinase fold. In contrast to the mutational activation of EGFR and BRAF kinases that are strongly associated with malignancies, mutations in the CDKs that cause cancers are relatively rare [26,27]. An intriguing question was how pathogenic mutations in CDK proteins are distributed in the catalytic core and whether the localization of mutational sites in these kinases may be related to the rigidity/flexibility distribution in these proteins. To explore these questions, we retrieved missense mutations for CDK5, CDK6 and CDK9 proteins from dbSAP collection [154] and COSMIC database [155]. Structural mapping of pathogenic mutations onto crystallographic conformations of studied CDK proteins was undertaken in the context of rigidity decomposition analysis and weak spot localization (Fig 11D–11F). A total of 21 CDK5 mutations, 28 CDK6 mutations and 37 CDK9 mutations were selected and mapped onto the crystal structures. This analysis indicated that the majority of pathogenic mutations were localized in the flexible regions and often targeted the positions occupied by the weak spots, where they can modulate conformational changes without perturbing the kinase fold. Another interesting trend showed that CDK5 mutations were localized mainly around the C-terminal flexible regions (Fig 11D). At the same time, pathogenic mutations in CDK6 (Fig 11E) and CDK9 clients (Fig 11F) occupied different flexible positions in both lobes. Of particular interest the presence of multiple pathogenic mutations in the N-lobe and C-terminal tail of CDK9. Using kinetic analysis of a human P-TEFb complex consisting of CDK9 and cyclin T, the experimental studies showed that the C-terminal tail in CDK9 is important for kinase activity and deletions in this region can change the kinetic mechanism [49]. Our results indicated that many mutational sites (including for example F336, E337 studied in [49]) occupied positions of weak spots in the CDK9 structure, suggesting that modifications in these regions can modulate functional motions by altering balance of rigidity and flexibility. Accordingly, mutations that disrupt the interactions in the flexible N-lobe and C-terminal tail of CDK9 may change the rigidity/flexibility balance and alter position of major weak spots. Through this mechanism, mutations may affect the global allosteric network and cause variations in the chaperone dependencies and regulatory responses of the kinase client. These factors may play an important role in phenotypic and functional plasticity of CDK9 that is associated with transcriptional control and can form distinct positive transcription elongation factors (P-TEFs). In addition, structural and dynamic adaptability of CDK9 is intimately connected with diverse therapeutic indications of this kinase client, as CDK9 inhibition contributes to the anticancer activity and is relevant for the treatment of inflammation-associated diseases [156].

To summarize, by exploring a battery of synergistic computational approaches, our results revealed the interplay between conformational dynamics, organization of the residue interaction networks and communication pathways in CDK proteins. These signatures can determine chaperone dependencies and differentiate kinase clients, providing helpful insights into divergences in the activation mechanisms of CDK family members. The results are also consistent with various structural and biochemical experiments, offering a simple and robust computational model to probe relationships between dynamics, allosteric and chaperone regulation of protein kinases.

## Conclusions

In this study, we reported the results of a computational investigation of several members of CDK family (CDK5, CDK6, CDK9) that represented a broad repertoire of chaperone dependencies—from nonclient CDK5, to weak client CDK6, and strong client CDK9. Despite adopting structurally similar active conformations in their respective complexes with cyclins and binding partners, these kinases have markedly different chaperone propensities and subtle differences in regulatory mechanisms. DMD and ENM approaches were used in conjunction with all-atom reconstruction to simulate dynamics of multiple crystal structures and characterize conformational ensembles of CDK5, CDK6, and CDK9 proteins. We found that the elevated dynamics of CDK9 can trigger imbalances in cooperative collective motions and reduce stability of the active fold, thus creating a cascade of favorable conditions for chaperone intervention. The ensemble-based modeling of residue interaction networks and community analysis determined how differences in modularity of allosteric networks and topography of communication pathways can be linked with the client status of CDK proteins. We also performed community decomposition and analyze modularity of the ensemble-averaged residue interaction networks in the CDK proteins. The results revealed a dense and stable interaction network in CDK5 that may be contrasted with a weaker and more fragmented network organization in CDK6 and CDK9 clients. We argue that this may result in the reduced cooperativity and compromise the efficiency of allosteric communication between functional regions needed for activation transitions, thereby leading to a divergence in the regulatory mechanisms. Our study provides evidence that strong client status of CDK9 protein may be linked with the elevated conformational mobility and reduced cooperativity that is induced by dynamic and energetic polarization of kinase lobes. We also employed perturbation response scanning analysis and rigidity-based decomposition to emulate thermal unfolding of CDK complexes. These approaches connected conformational dynamics and stability profiles with differences in allosteric communications and chaperone dependencies of CDK proteins. By investigating a panel of CDK proteins that span the complete spectrum of chaperone dependencies, we determined dynamic and network signatures that can differentiate kinase clients and rationalize subtle divergences in the activation mechanisms in the CDK family. Our study offers a simple and robust computational framework that links protein kinase dynamics and organization of allosteric interaction networks with molecular determinants underlying critical regulatory responses. This approach can be also useful in developing systems biology strategies for designing robust combinations of targeted and allosteric inhibitors of oncogenic kinase clients by interrogating and manipulating kinase preferences and binding affinities with the chaperone system.

## Materials and methods

### Discrete molecular dynamics and elastic network modeling

We employed the formalism of the discrete molecular dynamics (DMD) simulations [157–160] as implemented in [160] to simulate multiple crystal structures of CDK5, CDK6 and CDK9 proteins. The crystal structures of various CDK5-p25 complexes were simulated (pdb id 14H4L, 1UNG, 1UNH, 1UNL, 3O0G, 4AU8). The crystal structures of CDK6/V-cyclin complexes (pdb id 2EUF, 2F2C) and CDK6 complexes withy inhibitors (pdb id 5L2I, 5L2S, and 5L2T) were subjected to simulations. The simulated crystal structures of CDK9-cyclin T/T1 complexes included the following pdb entries: 3BLH, 3BLQ, 3BLR, 3LQ5, 3MIA, 3TN8, 3TNH, 4BCF, 4BCH, 4BCI, 4BCJ, 4EC8, 4EC9, 4IMY. In the DMD approach, the protein structures were modeled as systems consisting of *C*_*α*_ residue-based beads interacting through a discontinuous square well potential. In the basic DMD formalism [157] particles move in the ballistic regime under constant velocity until a collision between a pair of particles occurs at the distance where their pairwise potential energy changes, i.e. DMD consists of a sequence of atomic collisions. In the absence of any collision, the particles move linearly with constant velocity. The main advantage of DMD is elimination of time-consuming computations of forces and accelerations as compared to more demanding atomistic molecular dynamics MDs. [157,158]. In the DMD implementation used in our study, the interaction potentials are defined as infinite square wells, such that the particle-particle distances vary between *d*_min_ = (1-σ)r_ij_^0^ and *d*_min_ = (1+σ)r_ij_^0^ where r_ij_^0^ is the distance between particles (residues) *i* and *j* in the native conformation and 2σ the width of the square well. The MD-averaged conformation was taken as the native conformation. Residue-residue interaction potentials are defined for the particles at a distance smaller than a cut-off radius *r*_*c*_ in the native conformation. A small well width σ = 0.05 was used for neighboring particles to keep the *C*_*α*_—*C*_*α*_ distances closer to the expected equilibrium value of 3.8 Å. For nonconsecutive pairs of *C*_*α*_ particles, *r*_*c*_ = 8 Ǻ and σ = 0.1 were used. Using DMD simulations, we generated conformational landscapes of the CDK proteins in a coarse-grained representation. Conformational ensembles were then subjected to all-atom reconstruction using PULCHRA method [102] and CG2AA tool [103] that mapped atomistic structures from simulation trajectories obtained in a reduced protein representation. The reconstructed conformations were also optimized using the 3Drefine method [104,105] that utilizes atomic-level energy minimization with a composite physics and knowledge-based force fields.

The functional dynamics analysis of the Hsp90-cochaperone complexes was conducted using the GNM approach [161,162] in which protein structure is reduced to a network of *N* residue nodes identified by *C*_*α*_ atoms and the fluctuations of each node are assumed to be isotropic and Gaussian. The topology of the protein structure is described by *N*×*N* Kirchhoff matrix of inter-residue contacts **Г**, where the off-diagonal elements are −1, if the nodes are within a cutoff distance *r*_*c*_, and zero otherwise. Bonded and nonbonded pairs of residues located within an interaction cutoff distance *r*_*c*_ = 7.0 Å are assumed to be connected by springs with a uniform spring constant γ. GNM is used to compute the mobility profiles *M*_*i*_^(*k*)^ as a function of residue index *i*, for the normal mode *k*, as was presented in details in the original studies [163]. In GNM analysis, we considered the low frequency soft modes that can adequately describe global functional motions. The *i*^th^ element, [**u**^**(***k***)**^]_*i*_, of **u**^**(***k***)**^ describes the displacement of residue *i* along the *k*^th^ mode and ([**u**^**(***k***)**^]_i_)^2^ as a function of residue number *i* defines the mobility profiles *M*_*i*_^(*k*)^ along mode *k* [161,162]. The mobility profile averaged over a set of *m* modes is expressed as follows:

$${\langle M_{i}\rangle |}_{m}=\frac{\sum_{k=1}^{m}\lambda_{k}^{-1}{\left[u^{\left(k\right)}\right]}_{i}^{2}}{\sum_{k=1}^{m}\lambda_{k}^{-1}}=\frac{\sum_{k=1}^{m}\lambda_{k}^{-1}M_{i}^{\left(k\right)}}{\sum_{k=1}^{m}\lambda_{k}^{-1}}$$ (1)

Conformational mobility profiles in the space of low frequency modes were obtained using the iGNM [161] and ANM web servers [162]. In these profiles, the minima typically correspond to global hinge centers that control collective motions, while the peaks refer to more flexible regions involved in conformational transformations in functional dynamics.

### Ensemble-based residue interaction networks and modeling of communication pathways

A graph-based model of protein structure considers residues as network nodes while inter-residue edges represent residue interactions. The details of graph construction using residue interaction cut-off strength (*I*_min_) [95, 96] were outlined in our previous studies of molecular chaperones [164–166]. The edges in the residue interaction network are weighted based on dynamic residue correlations couplings obtained from MD simulations [98–100] and coevolutionary mutual information. Mutual Information (MI) analysis is used in the framework of MISTIC approach to estimate coevolutionary relationship between pairs of positions in the protein kinase family [126–128]. For this analysis, multiple sequence alignment (MSA) profile of the protein family was obtained from Pfam multiple sequence alignment (MSA) profile of the kinase family was obtained from Pfam database that includes accurate MSA of protein families generated using hidden Markov models [167–169]. The Kullback-Leibler (KL) sequence conservation score *KLConsScore* was also calculated using MSA profile of the protein kinase family with the aid of MISTIC server [126,127]. Sequences covering <50% of the reference sequence length were removed from MSA. For each column of the MSA, the KL conservation is calculated according to the following formula:

$$KLConsScore_{i}=\sum_{i=1}^{N}\text{ln}\frac{P(i)}{Q(i)}$$ (2)

Here, *P(i)* is the frequency of amino acid *i* in that position and *Q(i)* is the background frequency of the amino acid in nature calculated using an amino acids background frequency distribution obtained from the UniProt database.

In the network model of protein structures, weight *w*_*ij*_ is defined by the generalized correlation coefficient **r**_*MI*_ (**x**_i_,**x**_j_) measuring both dynamic and coevolutionary coupling between residue pairs [128]:

$$w_{ij}=-\text{log}{\left[r_{MI}(x_{i},x_{j})\right]}_{}$$ (3)

The length (i.e. weight) of the edge *w*_*ij*_ that connects nodes *i* and *j* is calculated using the generalized correlation coefficients **r**_*MI*_ (**x**_i_,**x**_j_) associated with the dynamic correlation and mutual information shared by each pair of residues [170,171]. The ensemble of shortest paths is determined from matrix of communication distances by the Floyd-Warshall algorithm [172] that compares all possible paths between each pair of residue nodes.

### Protein stability calculations

A systematic alanine scanning of protein residues in the CDK5 and CDK6 structures was performed using FoldX approach [117]. For this analysis, the FoldX force field was employed via graphical web-based interface [118] implemented as a plugin for the YASARA molecular graphics suit [119]. If a free energy change between a mutant and the wild type (WT) proteins ΔΔG = ΔG (MT)-ΔG (WT) > 0, the mutation is destabilizing, while when ΔΔG <0 the respective mutation is stabilizing. The protocol involved a systematic modification of the protein residues to alanine by eliminating side-chain atoms beyond *C*_β_, that is followed by 1,000 steps of steepest decent and Newton—Raphson minimizations to remove steric clashes and close contacts before calculating the energy terms and measuring the effect of alanine mutations on protein stability. Residues for which alanine mutations result in a significant destabilization effect (ΔΔG > 1.5–2.0 kcal/mol) can be regarded as potential energetic hot spots that are important for thermodynamic stability of the protein structures. Statistically significant average ΔΔG values were obtained from 100 independent samples of conformational ensembles for each studied structure [119]. Each of 100 independent samples used for ΔΔG estimates is formed by 1,000 conformations selected from 10 independent DMD simulations. This protocol allows to systematically evaluating the sample means and standard deviations of a population represented by 100 samples of 1,000 conformations each. We then computed the standard errors of the mean values <ΔΔG> derived from these populations and evaluated 95% confidence intervals for the reported <ΔΔG> values.

### Network centrality and community analysis

Using the constructed protein structure networks, we computed the residue-based betweenness parameter. The betweenness of residue *i* is defined to be the sum of the fraction of shortest paths between all pairs of residues that pass through residue *i*:

$$C_{b}(n_{i})=\sum_{j<k}^{N}\frac{g_{jk}(i)}{g_{jk}}$$ (4)

where *g*_*jk*_ denotes the number of shortest geodesics paths connecting *j* and *k*, and *g*_*jk*_*(i)* is the number of shortest paths between residues *j* and *k* passing through the node *n*_*i*_. Residues with high occurrence in the shortest paths connecting all residue pairs have a higher betweenness values. The residue betweenness values and the matrix of shortest communication pathways between residue pairs provide a measure of signaling flow passing through edges of the network that is used for network partition into local communities. Protein structure networks were initially analyzed for detection of *k*-cliques and *k*-clique communities using Clique Percolation algorithm [173] in which community is associated with a subgraph containing *k*-cliques that can be reached from each other through a series of adjacent *k*-cliques. We employed a community definition according to which in a *k*-clique community two *k*-cliques share *k*-1 or *k*-2 nodes. Computation of the network parameters was performed using the Clique Percolation Method as implemented in the CFinder program [174]. The communities that remained stable in more than 75% of the conformations in the equilibrium ensemble were reported and analyzed. The Girvan-Newmann algorithm [131–133] was used to maximize the modularity and optimize the quality of the community structure. This method utilizes the edge betweenness as a partitioning criterion and splits network into local communities, where the connections (interactions) within local communities are strong and dense, while the connections between communities are weaker and sparser. The Girvan-Newman algorithm is an iterative procedure in which the edge with the highest betweenness is removed from the network and the betweenness of the remaining edges is recalculated. The edge betweenness measures the importance of a particular connection in the global information flow of the network. This parameter is defined as the ratio of all the shortest paths passing through a particular edge to the total number of shortest paths in the network.

### Rigidity decomposition of the residue interaction networks

We utilized FIRST (Floppy Inclusion and Rigid Substructure Topography) approach [142–146] and the Python-based Constraint Network Analysis (CNA) interfaces [147,148] to build a network of the covalent and noncovalent bond constraints in the protein. In the FIRST approach, hydrogen bonds, salt bridges, and hydrophobic contacts are calculated using an empirical energy function. A hydrogen bond energy *E*_*hb*_ is calculated using a geometry-based empirical function [175] and only hydrogen bonds with an energy below cutoff *E*_*cut*,*hb*_ = -1.0 kcal/mol are included in the network. Hydrophobic contacts are considered between all carbon and sulfur atoms separated by a distance less than the sum of their van der Waals radii (1.7 Å for C and 1.8 Å for S) plus a temperature-independent *D*_*cut*,*hp*_ = 0.25 Å [176]. In the FIRST approach, rigidity changes are monitored by a gradual removal of hydrogen bonds in the order of increasing strength, keeping all covalent and hydrophobic interactions and repeating the rigidity analysis at each step, thus decomposing protein structure into rigid and flexible regions. Thermal unfolding in the FIRST approach is implemented by emulating temperature-dependent unfolding trajectories. During unfolding, non-covalent constraints corresponding to weaker interactions that dissolve at low temperatures are removed from the network first, and each new network is then again decomposed into rigid and flexible clusters. By proceeding from a rigid network at low temperature to a flexible network at high temperature, unfolding phase transitions can be observed, at which point a giant rigid cluster in the network breaks apart into smaller rigid clusters. The identification of weak spots is performed using the CNA software package and webserver [147,148]. In this procedure, rigid cluster decompositions immediately before and after folded-unfolded transition are compared, and residues whose C_α_ atoms are part of the giant cluster before the transition, and leave the giant cluster after transition are identified as locally weak spots in the constraint network. A residue is considered flexible if its C_α_ atom is either in a flexible region or part of a small rigid cluster of less than four atoms. In our application of the FIRST approach, we considered conformational ensembles of multiple crystal structures of CDK proteins. The identification of weak spots is carried out for 1,000 representative conformations of the ensemble for each of the crystal structure. The frequencies of residues to become weak spots are computed and averaged over conformational ensembles of multiple crystal structures for each studied CDK protein. The frequency of all residues being predicted as a weak spot throughout the ensemble is counted and, finally, all weak spots are assigned a rank according to the decreasing order of their frequency.

## Supporting information

S1 Fig Structure-based survey of the protein kinase clients. Kinome mapping of Hsp90-Cdc37 clients extracted from experimental studies [72–74] is depicted (A). The kinases that are found to be downregulated by Hsp90 inhibition in the experimental profiling are shown in yellow (confirmed kinase clients) and red (novel kinase clients discovered in [73]). (B) Structure-based kinome mapping of the Hsp90-Cdc37 kinase clients. The Cdk/Src kinase clients are marked in blue filled spheres. A high density of the Cdk/Src clients in the TK, TKL, STE, CAMK, and CMGC groups of the human kinome tree is highlighted by blue circles. The second category of kinase clients is characterized by active structures stabilized through allosteric interactions with regulatory motifs (marked in green spheres). A noticeable presence of these kinase clients in the AGC group of kinases is highlighted by the green circle.
(TIF)

S2 Fig Sequence conservation profiles of CDK proteins. The Kullback-Leibler (KL) conservation score is mapped onto respective kinase residues in the crystal structures of CDK5 (A), CDK6 (B) and CDK9 proteins (C). The KL profiles are shown in red bars for CDK5 (A), green bars for CDK6 (B) and blue bars for CDK9 residues (C). Sequence conservation of critical functional regions HRD and DFG is highlighted by filled marron diamonds. Sequence mapping onto crystal structures residues is undertaken to facilitate direct comparison with conformational dynamics and structural stability of CDK proteins.
(TIF)

S3 Fig Analysis of the residue interaction networks and hubs of allosteric communications in the CDK5 mutational variants. Residue-based centrality distributions of the CDK5-F145A mutant (A) and CDK5-S159A mutant (B). The network profile of the WT CDK5-p25 (pdb id 3O0G) is shown in (A) and (B) in filled brown bars as a reference for comparison with the centrality profiles of the mutants. The centrality distributions for CDK5-F145A mutant and CDK5-S159A mutant are shown in marron bars. The distributions are derived by averaging computations of network parameters over the conformational ensembles obtained from DMD simulations of CDK5 mutants. Structural mapping of high centrality edges in the CDK5-F145A mutant complex (C) and in the CDK5-S159A complex (D). The kinase domains are shown in green ribbons and P25 protein is shown in cyan ribbons. The residues forming high centrality edges are shown in red spheres.
(TIF)
