# Supplementary figures and images for: Ensemble-based modeling and rigidity decomposition of allosteric interaction networks and communication pathways in cyclin-dependent kinases: Differentiating kinase clients of the Hsp90-Cdc37 chaperone
Source: PLoS One. 2017 Nov 2;12(11):e0186089. doi: 10.1371/journal.pone.0186089 (PMC5667858; doi:10.1371/journal.pone.0186089)

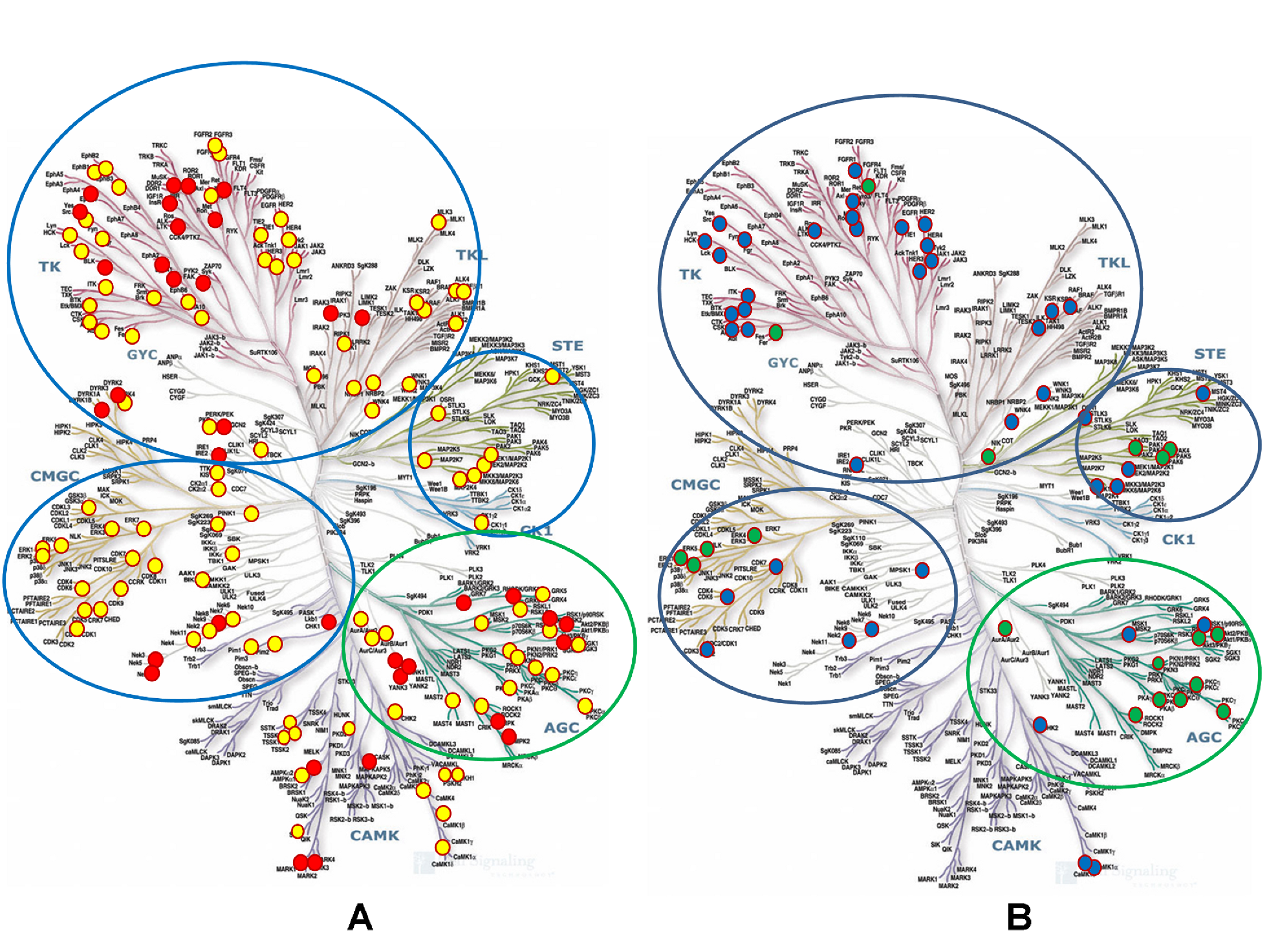

Supplement: S1 Fig — Kinome mapping of Hsp90-Cdc37 clients extracted from experimental studies [72–74] is depicted (A). The kinases that are found to be downregulated by Hsp90 inhibition in the experimental profiling are shown in yellow (confirmed kinase clients) and red (novel kinase clients discovered in [73]). (B) Structure-based kinome mapping of the Hsp90-Cdc37 kinase clients. The Cdk/Src kinase clients are marked in blue filled spheres. A high density of the Cdk/Src clients in the TK, TKL, STE, CAMK, and CMGC groups of the human kinome tree is highlighted by blue circles. The second category of kinase clients is characterized by active structures stabilized through allosteric interactions with regulatory motifs (marked in green spheres). A noticeable presence of these kinase clients in the AGC group of kinases is highlighted by the green circle. (TIF) [file pone.0186089.s001.tif]

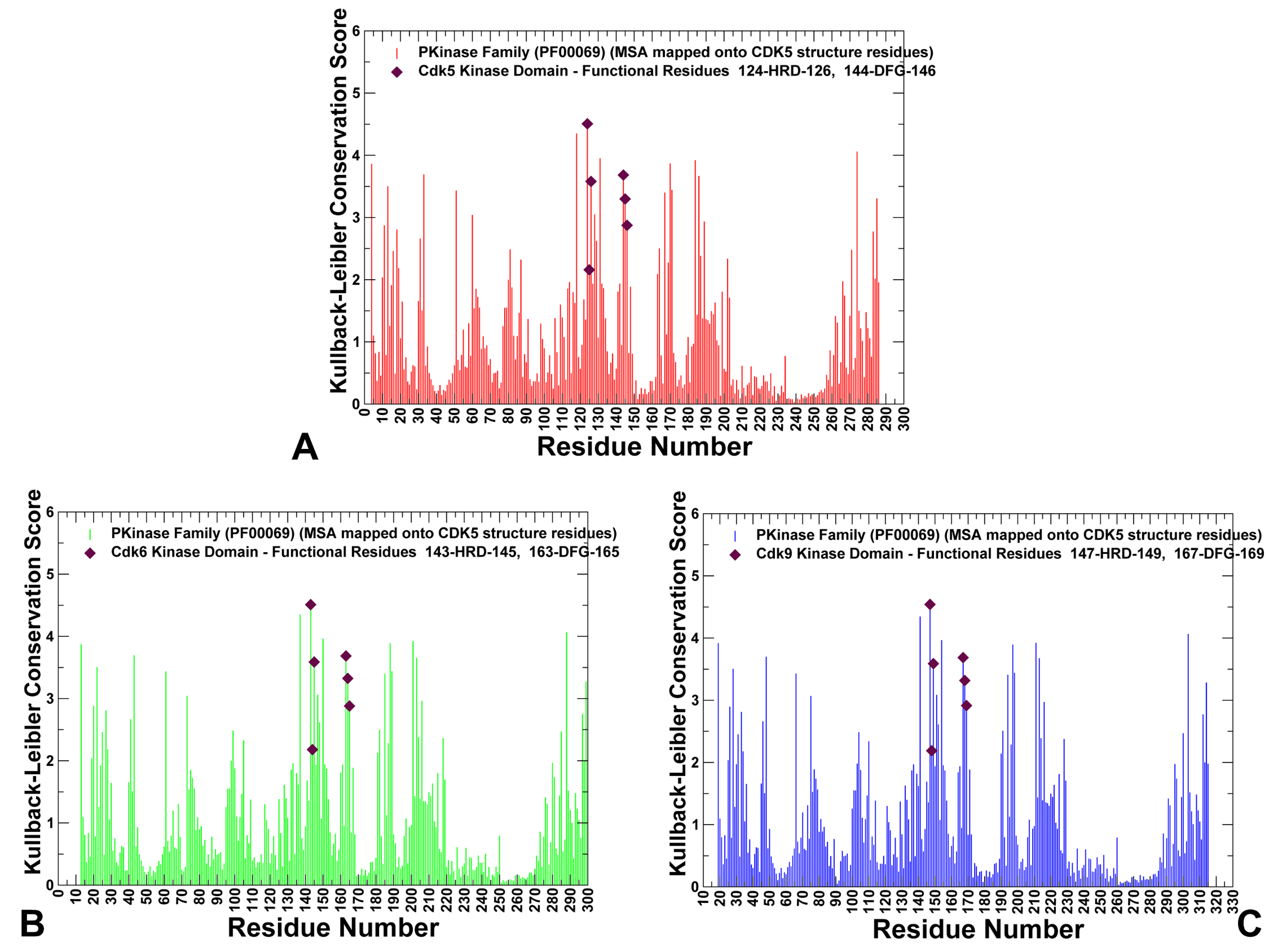

Supplement: S2 Fig — The Kullback-Leibler (KL) conservation score is mapped onto respective kinase residues in the crystal structures of CDK5 (A), CDK6 (B) and CDK9 proteins (C). The KL profiles are shown in red bars for CDK5 (A), green bars for CDK6 (B) and blue bars for CDK9 residues (C). Sequence conservation of critical functional regions HRD and DFG is highlighted by filled marron diamonds. Sequence mapping onto crystal structures residues is undertaken to facilitate direct comparison with conformational dynamics and structural stability of CDK proteins. (TIF) [file pone.0186089.s002.tif]

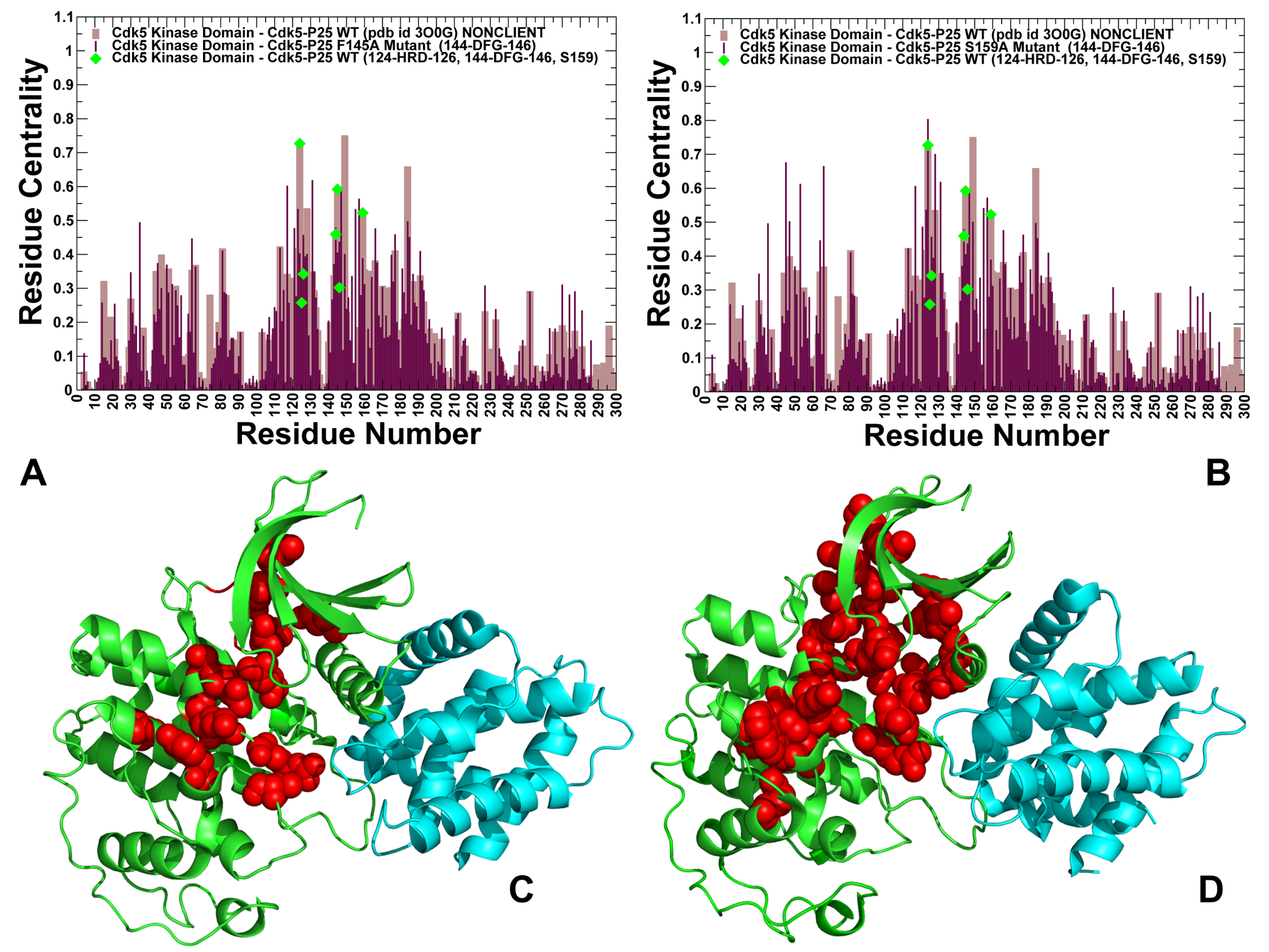

Supplement: S3 Fig — Residue-based centrality distributions of the CDK5-F145A mutant (A) and CDK5-S159A mutant (B). The network profile of the WT CDK5-p25 (pdb id 3O0G) is shown in (A) and (B) in filled brown bars as a reference for comparison with the centrality profiles of the mutants. The centrality distributions for CDK5-F145A mutant and CDK5-S159A mutant are shown in marron bars. The distributions are derived by averaging computations of network parameters over the conformational ensembles obtained from DMD simulations of CDK5 mutants. Structural mapping of high centrality edges in the CDK5-F145A mutant complex (C) and in the CDK5-S159A complex (D). The kinase domains are shown in green ribbons and P25 protein is shown in cyan ribbons. The residues forming high centrality edges are shown in red spheres. (TIF) [file pone.0186089.s003.tif]
